# Epithelial apical glycosylation changes associated with thin endometrium in women with infertility - a pilot observational study

**DOI:** 10.1186/s12958-021-00750-z

**Published:** 2021-05-15

**Authors:** Marina M. Ziganshina, Nataliya V. Dolgushina, Galina V. Kulikova, Nafisa M. Fayzullina, Ekaterina L. Yarotskaya, Nailia R. Khasbiullina, Nigora F. Abdurakhmanova, Aleksandra V. Asaturova, Alexander I. Shchegolev, Alina A. Dovgan, Gennady T. Sukhikh

**Affiliations:** 1 Laboratory of Clinical Immunology, National Medical Research Center for Obstetrics, Gynecology, and Perinatology named after Academician V.I. Kulakov of the Ministry of Healthcare of Russian Federation, Oparina str. 4, Moscow, Russian Federation 117997; 2 R&D Department, National Medical Research Center for Obstetrics, Gynecology, and Perinatology of Ministry of Healthcare of Russian Federation, Oparina str. 4, Moscow, 117997 Russia; 3 First Moscow State Medical University named after I.M. Sechenov, Trubetskaya str. 8-2, Moscow, 119991 Russia; 4 Department of Pathology, National Medical Research Center for Obstetrics, Gynecology, and Perinatology of Ministry of Healthcare of Russian Federation, Oparina str. 4, Moscow, 117997 Russia; 5 Department of International Cooperation, National Medical Research Center for Obstetrics, Gynecology, and Perinatology of Ministry of Healthcare of Russian Federation, Oparina str. 4, Moscow, 117997 Russia; 6 Department of Assisted Technologies in Treatment of Infertility, National Medical Research Center for Obstetrics, Gynecology, and Perinatology of Ministry of Healthcare of Russian Federation, Oparina str. 4, Moscow, 117997 Russia

**Keywords:** Thin endometrium, Infertility, Assisted reproductive technologies (ART), Lectin histochemistry, Glycans, Glycocalyx, MECA-79

## Abstract

**Background:**

Low endometrial receptivity is one of the major factors affecting successful implantation in assisted reproductive technologies (ART). Infertile patients with thin endometrium have a significantly lower cumulative clinical pregnancy rate than patients with normal endometrium. Molecular pathophysiology of low receptivity of thin endometrium remains understudied. We have investigated composition of glycocalyx of the apical surface of luminal and glandular epithelial cells in thin endometrium of infertile women.

**Methods:**

Thirty-two patients with tubal-peritoneal infertility undergoing in vitro fertilization (IVF) were included in the study. Endometrial samples were obtained in a natural menstrual cycle. Patients were divided into two groups: patients with normal endometrium (≥8 mm) and with thin endometrium (< 8 mm). Histochemical and immunohistochemical analysis of paraffin-embedded endometrial samples was performed using six biotinylated lectins (UEA-I, MAL-II, SNA, VVL, ECL, Con A) and anti-Le^Y^ and MECA-79 monoclonal antibodies (MAbs).

**Results:**

Complex glycans analysis taking into account the adjusted specificity of glycan-binding MAbs revealed 1.3 times less expression of MECA-79 glycans on the apical surface of the luminal epithelial cells of thin endometrium compared to normal endometrium; this deficiency may adversely affect implantation, since MECA-79 glycans are a ligand of L-selectin and mediate intercellular interactions. The glycans containing a type-2 unit Galβ1-4GlcNAcβ (LacNAc) but lacking sulfo-residues at 6-OH of GlcNAcβ, and binding to MECA-79 MAbs were found; they can be considered as potential markers of endometrium receptivity.

Expression of the lectins-stained glycans on the apical surfaces of the luminal and glandular epithelial cells did not differ significantly. Correlation between the expression of difucosylated oligosaccharide Le^Y^ on the apical surfaces of the luminal and glandular epithelial cells was found in patients with thin endometrium and recurrent implantation failure. A similar relationship was shown for mannose-rich glycans.

**Conclusions:**

Specific features of key glycans expression in epithelial compartments of thin endometrium may be essential for morphogenesis of the endometrial functional layer and explain its low receptivity.

**Supplementary Information:**

The online version contains supplementary material available at 10.1186/s12958-021-00750-z.

## Background

Endometrial thickness was proposed to be a prognostic criterion for pregnancy by Gonen et al. in 1989 [1]. Since then, the concept of minimal valid endometrial thickness was actively used in clinical practice. Endometrium less than 6–8 mm is now considered to be “thin” [2–4]. A predictive value of this indicator is contradictory, since pregnancies have been reported to occur in patients with endometrium 4–5 mm, suggesting that receptivity might be not necessarily related to endometrial thickness [5, 6]. However, many experts agree that insufficient endometrial thickness may reduce the cumulative clinical pregnancy rate, live birth rate [7], and be associated with small for gestational age neonates and obstetric complications caused by poor placentation [8].

Current data on molecular changes in thin endometrium, reflecting pathophysiological mechanisms of endometrial receptivity reduction [9–11] are very limited. Endometrial receptivity can be only assessed in non-conception cycles, where endometrial thickness and receptivity may vary. Therefore, a complex assessment of the molecular composition of the main functional structures of endometrium in a natural cycle is needed. This could allow prediction of the endometrium potential for blastocyst acceptance and trophoblast invasion. Molecular research should be obviously focused on the key molecules involved in intercellular interactions mediating these processes.

Human endometrium, where most proteins on the cellular surface are glycosylated, demonstrate particularly rich in glycans expression [12, 13]. Glycoconjugates are associated with the apical surfaces of epithelial cells, which contact with blastocyst during implantation. Secretions of the endometrial glands contain a lot of glycoproteins and mucins that promote specific uterine functions [14, 15]. However, even though the studies of glycans associated with the apical surface of luminal and glandular epithelial cells have been performed earlier [15–19], there is no data on the composition of glycocalyx of thin endometrium.

The analysis of functional carbohydrate residues, which are important part of glycoproteins, glycolipids, and extracellular matrices of endometrial tissue, may reveal the carbohydrate phenotype (the “glycocode”) of thin endometrium. Detection of the coherent changes of glycans in the endometrial epithelial structures may determine their importance for normal and thin endometrium and their impact on receptivity. In this regard, the study was aimed to evaluate complex changes in the composition of glycocalyx and to analyze the expression of glycans in luminal and glandular epithelial cells of normal and thin endometrium in patients with infertility.

## Methods

### Patients and endometrial biopsy samples

Thirty-two patients with tubal-peritoneal infertility undergoing IVF treatment were included in the study. Endometrial samples were obtained in a natural menstrual cycle around day 6 after ovulation (cycle day 20–22, «implantation window»). The patients did not receive any hormonal therapy for at least 2 months before the procedure. All samples were examined by the same pathologist using the dating criteria described by Noyes RW [20].

In our previous study [21] of 154 infertile patients stratified into 2 groups (43 patients with endometrial thickness 9.3 ± 0.9 mm who achieved pregnancy by IVF, and 110 patients with endometrial thickness 7.2 ± 0.9 mm, who failed to conceive (*p* < 0.0001)), we have found that a threshold of endometrial thickness, with which the odds of pregnancy and the validity of the logistic regression model increased, was 8.0 mm (AUC = 86.7%, Se =97.7%, Sp =75.7%). Basing on this observation, for the current study we have set the threshold of endometrial thickness 8 mm.

Depending on the endometrium thickness, the patients were divided into two groups: patients with “thin” endometrium (< 8 mm, *n* = 14), group 1, and patients with normal endometrium (≥8 mm, *n* = 18), group 2.

Inclusion criteria were normal karyotypes of the patient and her partner; age 18–40 years; body mass index (BMI) 18–29.9 kg/m^2^. Exclusion criteria were: contraindications to IVF; pathozoospermia in the partner; use of donor gametes or surrogacy; poor ovarian response to stimulation; absence of good quality blastocysts; and IVF complications in the studied cycle.

The IVF program (the next month after endometrial biopsy) involved ovarian stimulation with recombinant follicle-stimulating hormone (rFSH) or a combination of rFSH and luteinizing hormone (LH) and treatment with gonadotropin-releasing hormone antagonists (GnRH-ant). The ovulation trigger (HCG 8.000–10.000 IU) was administered when leading follicles reached 17 mm or more in diameter. Transvaginal puncture (TVP) was performed 36 h after the administration of the ovulation trigger.

Oocytes were fertilized by intracytoplasmic sperm injection (ICSI), because of subfertility of the male partner and low fertilization rate in the previous IVF cycle. Morphological evaluation of the embryos was performed on the 5th day after TVP, according to Gardner et al. classification [22].

The embryo transfer (ET) was performed on the 5th day after TVP in a fresh cycle. One blastocyst of the best quality was transferred into the uterine cavity. Biochemical pregnancy was stated if the level of serum ß-HCG increased 14 days after ET; a clinical pregnancy was stated if ultrasound scan showed the gestational sac 21 days after ET.

### Antibodies

Purified mouse anti-blood group Lewis^Y^ (Le^Y^) monoclonal antibodies, clone LWY/1463, Abcam, ab 219,336, 1/200 (anti-Le^Y^ MAbs) and purified rat anti-mouse peripheral lymph node addressin (PNAd) carbohydrate epitope monoclonal antibodies, clone MECA-79, BD Pharmingen, Cat. No 553863, 1/100 (MECA-79 MAbs) were used to determine the expression of the glycans epitope in the endometrium. The anti-Le^Y^ MAbs recognize a difucosylated oligosaccharide Le^Y^ (major specificity Fucα1–2Galβ1–4(Fucα1–3)GlcNAcβ-R). MECA-79 MAbs react with carbohydrate epitopes which are a high-affinity L-selectin ligand (CD34, GlyCAM-1, MAdCAM-1). As previously reported, MECA-79 epitope contain *(*6-O-Su)GlcNAcβ-residue [23].

### Lectins

A panel of six selected biotinylated lectins (Vector Labs, USA) was used to study the glycocalyx composition in the structural elements of the endometrium: MAL-II (*Maackia amurensis* lectin-II, binding specificity NeuNAcα2-3Galβ); SNA (*Sambucus nigra* lectin, binding specificity NeuNAcα2-6Gal/GalNAc); ECL (*Erythrina cristagalli* lectin, binding specificity Galβ1-4GlcNAcβ (LacNAc, type-2 unit)); UEA-I (*Ulex europaeus* agglutinin-I, binding specificity H type 2 antigen L-Fucα1-2Galβ1-4GlcNAcβ1 and difucosylated oligosaccharide Le^Y^); VVL (*Vicia villosa* lectin, binding specificity GalNAcα1-Ser/Thr and Galα1-3GalNAc); Con A (Сoncanavalin A from *Canavalia ensiformis*, binding specificity D-Glcα and D-Manα (terminal or 1–2-linked) in high mannose, intermediate and small complex N-linked sequences) [24]. An optimal concentration for each lectin from the panel, which allowed the maximum labeling with minimum non-specified background, was 10 μg/ml for MAL-II and VVL, 5 μg /ml for UEA-1, and 2,5 μg/ml for lectins SNA, ECA, and Con A.

### Lectin histochemistry and immunohistochemistry

For lectin histochemistry, the paraffin sections, after dewaxing and hydration, were treated with 3% hydrogen peroxide for 10 min to inhibit the endogenous peroxidase, rinsed with distilled water, and treated with an avidin-biotin kit (Vector Labs, USA) to block endogenous avidin-binding activity. The sections were rinsed with phosphate-buffered saline (PBS) and incubated with Carbo-Free block solution (Vector Labs, USA) for 30 min to reduce the background staining. Then the sections were incubated for 30 min at room temperature with biotinylated lectins solution in PBS. After washing the sections were incubated with the avidin-biotin-peroxidase complex (ABC, Vector Labs, USA) for 30 min. After 3 washes in PBS, the peroxidase activity sites were visualized using DAB solution.

For immunohistochemistry, endometrial samples were fixed in 10% buffered formaldehyde for 24 h, embedded in paraffin, and 3 μm thick sections were prepared for immunohistochemical (IHC) investigation. IHC staining of citrate-pretreated sections (90 for 20 min) was performed using MAbs: anti-Le^Y^ MAbs (1 μg/ml) and MECA-79 MAbs (5 μg/ml). The binding of Le^Y^ was detected by Dako REAL™ EnVision™ Detection System (Dako, Denmark). The binding of MECA-79 MAbs was revealed with the use of a goat anti-rat IgG (H + L) (1 μg/ml, Vector Laboratories, USA), conjugated with peroxidase. Slides were counterstained with hematoxylin, dehydrated, cleared, and mounted in Shandon-mount to be examined under light microscopy.

### Controls for lectin and MAbs staining

Negative controls were carried out by replacement of the lectins and MAbs solution with a buffer (PBS, pH 7.4) to identify non-specific binding of avidin–peroxidase to the tissue or residual endogenous peroxidase activity. An irrelevant rat IgM antibody (purified rat IgM, κ isotype control clone R4-22, BD Pharmingen, Cat. No 553940), and mouse IgG1 (κ isotype control 15-6E10A7, Abcam, ab 170190) MAbs were used instead the primary antibody. Sections were also incubated with lectins in the presence of an appropriate competing sugar: 0.2 M α-methyl mannoside for Con A, 0.2 M L-fucose for UEA-1, 0.2 M galactose for ECL, 0.2 M N-acetylgalactosamine for VVL. The lung carcinoma, breast cancer lymph nodes and placental terminal villi were used as positive controls for staining of endometrium with an anti-Le^Y^ antibody, MECA-79 antibody and with lectins ConA and SNA, respectively.

### Neuraminidase digestion

For lectins ECL, SNA, and MAL-II, sialic acid was removed by pretreating the sections with 50 mU/ml neuraminidase (N) from *Vibrio cholerae* (Type III, Sigma-Aldrich, USA). Neuraminidase pre-treatment was used for 2 h at 37 °C, as described by Jones CJP [25].

### Assessment of staining location and reactivity intensity

A grid, 1 mm × 1 mm, divided into 100 fields, was used for staining assessment. The reaction intensity was assessed by two independent researchers “blindly” (the samples were encrypted). In each examination 10 randomly selected equidistant fields of view were studied using a random number generator. The measurements were processed automatically as average values of optical density on the apical surface of luminal epithelial cells and glandular epithelial cells using image analysis with NIS Elements Advanced Research 3.2 program (Laboratory Imaging LTD, Czech Republic).

### Microarray chip analysis

The microarray applied in the study was a polymer-coated glass slide with N-hydroxysuccinimide-derivatized surface, produced by Schott-Nexterion (Germany), with 651 spacered glycans in 50 μM solutions at 6 replicates [26]. A complete list of printed glycans is presented in an additional file (Table S1). A sample of anti- Le^Y^ MAbs or MECA-79 MAbs (both at concentration 5 μg/mL), diluted in PBS (Sigma Aldrich, USA) containing 0.1% (v/v) of Tween 20 (Merck, USA) and 1% BSA (Sigma, USA), was placed onto the chip surface and incubated in a humidified dark chamber at 37 C and mild rotation (32–34 rot/min) for 1 h. Then the chip was washed with PBS containing 0.05% Tween 20 (PBS-0.05%) and incubated with biotinylated goat-anti-mouse antibodies (Thermo Fisher Scientific, USA) at concentration 10 μg/ml in PBS-0.1% under the same conditions. Then the chip was washed with PBS-0.05% and incubated with streptavidin-Alexa-555 conjugate (Thermo Fisher Scientific, USA) at 1 μg/ml in PBS-0.1% at room temperature (20 °C) for 45 min and mild rotation. The chip was air-dried and scanned by InnoScan 710 AL (Innopsys, France) at λ_ex_ 543 nm and 10 μm resolution. The obtained data were processed using ScanArray Express 4.0 software, a fixed 80 μm-diameter circle method and Microsoft Excel software. Data for each glycan on the array are reported as median relative fluorescence units (RFU) of 6 replicates. The median deviation was measured as an interquartile range. A signal with fluorescence intensity 5 times exceeding the background value was considered significant.

### Statistical analysis

A statistical software package Statistica 10 (USA) was used to perform a χ^2^ test to compare categorical variables, t-test, linear regression, or Mann–Whitney U test. The odds ratio (OR) adjusted for confounders and calculated using logistic regression was used as a measure of association to compare binary data. Correlation analysis was performed with the use of Spearman’s rank correlation coefficient. Differences between statistical values were considered statistically significant at a confidence level *p*<0.05.

## Results

### Clinical results

The average age and BMI of patients in the studied groups did not differ significantly (p>0.05). There were also no differences in menstrual function, gravidity, parity, hormonal tests, and somatic morbidity. Patients with thin endometrium often had a history of endometrial polyps. There were no differences in cumulative doses of gonadotropins (GT), duration of ovarian stimulation, or in drugs used for ovarian stimulation (Table 1). A larger number of excellent blastocysts were obtained from patients with normal endometrial thickness (Table 2).

**Table 1:** Selected characteristics of the patients, included in the study

Features	Group 1 (*n* = 14)	Group 2 (*n* = 18)	*p*-value
Age, years^b^	33.0 ± 5.5	33.3 ± 3.3	0.8611
BMI, kg/m^2b^	22.4 ± 3.0	22.5 ± 2.6	0.9267
Endometrial polyps ^a^	5 (35.7%)	2 (11.1%)	0.0948
Gravidity^c^	0 (0–1)	1 (0–2)	0.2706
Parity^c^	0 (0–0)	0 (0–0)	0.6622
Number of IVF cycles^c^	0 (0–2)	1 (0–2)	0.4250
FSH, mIU/mL^b^	7.4 ± 2.8	7.8 ± 2.2	0.5781
AMH, ng/mL ^b^	3.5 ± 3.0	4.1 ± 3.9	0.6377
Stimulation duration, days^b^	8.9 ± 0.7	9.2 ± 1.3	0.5432
GT cumulative dose, IU^b^	1419.6 ± 371.6	1612.5 ± 532.3	0.2581

*BMI* body mass index, *FSH* follicle-stimulating hormone, *AMH* anti-Mullerian hormone, *GT* gonadotropins

^a^ Data are presented as absolute numbers and %, χ^2^ test

^b^ Data are presented as mean ± standard deviation, t-test

^c^ Data are presented as medians (with interquartile range), Kruskal-Wallis test

**Table 2:** Characteristics of gametes and embryos of the patients included in the study

Feature	Group 1 (*n* = 14)	Group 2 (*n* = 18)	p-level
Normal sperm ^a^	8 (57.1%)	14 (77.7%)	0.3193
Average number of mature oocytes per patient ^b^	6 (3–7)	4 (4–7)	0.7612
Average number of zygotes per patient ^b^	6 (2–7)	4 (3–6)	0.6622
Average fertilization rate ^c^	0.72 ± 0.15	0.75 ± 0.23	0.7648
Average number of blastocyst per patient^b^	4 (1–5)	3 (2–4)	0.9848
Average level of blastulation^c^	0.68 ± 0.26	0.77 ± 0.26	0.3143
Average number of excellent blastocysts per patient ^b^	0 (0–2)	1.5 (1–3)	0.0303

^a^ Data are presented as absolute numbers and %, χ2 test

^b^ Data are presented as medians (with interquartile range), Mann–Whitney U test;

^c^ Data are presented as mean ± standard deviation, t-test

The pregnancy rate in patients with endometrial thickness ≥ 8 mm was 66.7% (12 out of 18 women), while patients with thin endometrium failed to become pregnant (0 out of 14) (*p* = 0.0001). In the multivariate analysis with logistic regression, taking into account the number of excellent blastocysts, the OR of pregnancy, depending on the endometrium thickness, was 1.47 (95% CI = 1.05; 2.5).

### Intragroup comparison and correlation analysis of the glycans expression levels on the apical surface of the luminal and glandular epithelial cells in women with normal and thin endometrium

**Fig. 1:**
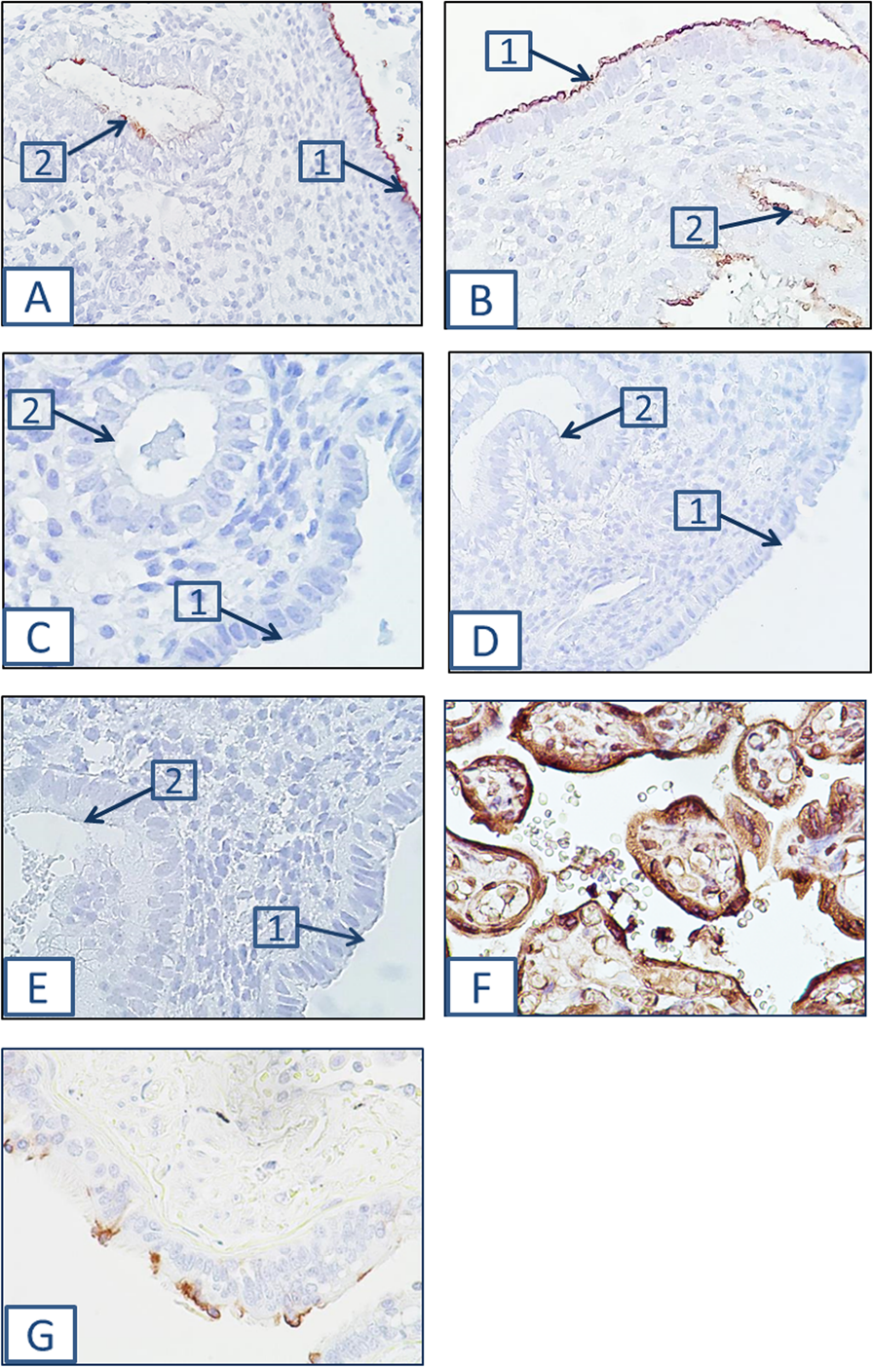
Glycoconjugates staining in thin endometrium. Staining of the apical surface of the luminal epithelial cells (1) and of the glandular cells (2) with anti-Le^Y^ MAbs (**a**) and with Сon A (**b**). Negative controls were performed without primary antibodies (**c**) and lectin Con A (**d**) and with mouse IgG_1_ (**e**). Staining of positive controls: **f** - placental terminal villi with Con A, **g** - lung carcinoma tissues with the anti-Le^Y^ antibody. Magnification: × 400

The glycans expression on the apical surfaces of luminal and glandular epithelial cells was assessed, and correlations between the expression levels were calculated for each studied group separately. The data obtained for each group were analyzed thereafter to find out the patterns of the glycans expression in normal and thin endometrium. In both groups, the glycans expression patterns were similar in both endometrial structures, as confirmed by correlation and comparative analysis with linear regression (Tables 3 and 4). In case of thin endometrium, a moderate positive correlation was observed for the contents of glycoconjugates stained with anti-Le^Y^ MAbs (an example is one of the photomicrographs of Fig. 1a) and Con A (Fig. 1b), UEA-I, N + ECL on the apical surface of the luminal epithelial cells, and the contents of the same glycoconjugates on the apical surface of glandular cells (Table 3). In patients with normal endometrium, a moderate positive correlation was found for staining with lectins UEA-I and N + ECL in the two endometrial structures (Table 4). It should be noted that despite the revealed significant relationship between the expression of subterminal residues Galβ1–4GlcNAcβ1- (N + ECL) in the studied endometrial structures of patients of both groups, their contents alterations were reciprocal, in contrast to the above pattern. The comparative results of lectins staining in the glycocalyx of luminal and glandular epithelial cells of endometrium in the studied groups are shown in Tables 3 and 4.

**Table 3:** Glycoconjugates contents on the apical surface of the luminal and glandular epithelial cells in thin endometrium

Lectins and MAbs	Luminal cells	Glandular cells	r_s_	*p*-value
MAL II	0.29 ± 0.07 (0.18–0.41)	0.27 ± 0.08 (0.15–0.45)	0.3475	0.1717
N + MAL II	0.16 ± 0.04 (0.09–0.24)	0.10 ± 0.03 (0.05–0.17)	0.4510	0.0692
UEA I	0.24 ± 0.11 (0.06–0.50)	0.17 ± 0.10 (0.08–0.41)	0.6472	***0.0036***
SNA	0.34 ± 0.09 (0.19–0.48)	0.22 ± 0.08 (0.10–0.38)	0.2675	0.2832
N + SNA	0.32 ± 0.09 (0.14–0.44)	0.17 ± 0.07 (0.07–0.29)	0.5697	***0.0135***
ECL	0.35 ± 0.12 (0.11–0.51)	0.26 ± 0.10 (0.10–0.42)	0.0734	0.7720
N + ECL	0.51 ± 0.11 (0.24–0.68)	0.57 ± 0.28 (0.27–1.49)	0.5873	***0.0103***
VVL	0.25 ± 0.10 (0.15–0.46)	0.24 ± 0.13 (0.07–0.53)	0.3771	0.1229
Con A	0.39 ± 0.09 (0.26–0.53)	0.28 ± 0.06 (0.17–0.44)	0.5064	***0.0319***
MECA-79	0.33 ± 0.07 (0.25–0.48)	0.19 ± 0.07 (0.03–0.29)	0.1019	0.7072
Anti- Le^Y^	0.25 ± 0.06 (0.15–0.39)	0.14 ± 0.06 (0.08–0.34)	0.4791	***0.0442***

Data are presented as mean ± standard deviation (minimum-maximum)

r_s_ Spearman’s rank correlation coefficient between contents of glycoconjugates in luminal and glandular epithelial cells

*P*-value for the Spearman’s rank statistic

*Italics Bold* statistically significant values

**Table 4:** Glycoconjugates contents on the apical surface of the luminal and glandular epithelial cells in normal endometrium

Lectins and MAbs	Luminal cells	Glandular cells	r_s_	*p*-value
MAL II	0.28 ± 0.11 (0.14–0.52)	0.27 ± 0.08 (0.15–0.45)	0.2100	0.4711
N + MAL II	0.15 ± 0.05 (0.10–0.30)	0.12 ± 0.08 (0.04–0.35)	0.0336	0.9093
UEA I	0.24 ± 0.14 (0.07–0.59)	0.22 ± 0.14 (0.05–0.49)	0.5925	***0.0328***
SNA	0.41 ± 0.07 (0.22–0.51)	0.22 ± 0.09 (0.09–0.41)	0.5040	0.0661
N + SNA	0.34 ± 0.08 (0.21–0.52)	0.19 ± 0.11 (0.06–0.46)	0.1990	0.4951
ECL	0.33 ± 0.09 (0.15–0.52)	0.29 ± 0.14 (0.08–0.62)	0.3812	0.1786
N + ECL	0.57 ± 0.09 (0.39–0.78)	0.55 ± 0.10 (0.39–0.72)	0.5697	***0.0334***
VVL	0.26 ± 0.09 (0.10–0.43)	0.21 ± 0.09 (0.09–0.38)	0.5294	0.0628
Con A	0.35 ± 0.09 (0.15–0.54)	0.30 ± 0.07 (0.17–0.40)	0.1505	0.6236
MECA-79	0.42 ± 0.11 (0.19–0.65)	0.20 ± 0.06 (0.10–0.31)	0.4476	0.1445
Anti- Le^Y^	0.22 ± 0.06 (0.12–0.35)	0.14 ± 0.06 (0.05–0.27)	−0.2903	0.3139

Data are presented as mean ± standard deviation (minimum-maximum)

r_s_ Spearman’s rank correlation coefficient between contents of glycoconjugates in luminal and glandular epithelial cells

*P* value for the Spearman’s rank statistic

*Italics Bold* statistically significant values

### Intergroup comparison of the glycans expression on the apical surface of the luminal and glandular epithelial cells in women with normal and thin endometrium

**Fig. 2:**
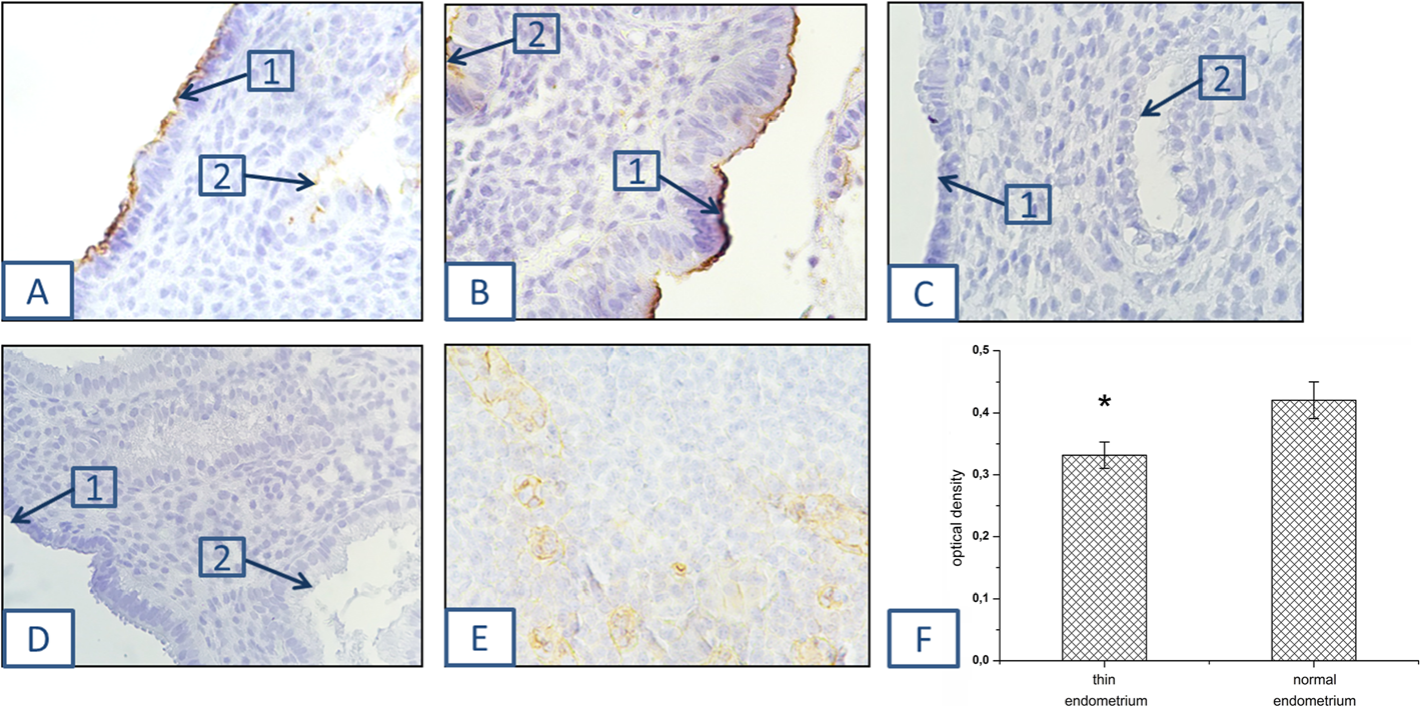
Immunolocalization of carbohydrate epitopes MECA-79 on the apical surface of the luminal epithelial cells. **a** - thin and **b** - normal endometrium; (1) - luminal cells and (2) - glandular cells. Negative controls were performed without primary antibodies (**c**) and with isotypic rat IgM (**d**). Positive control - lymph nodes in breast cancer (**e**). Magnification: × 400. Histograms demonstrate relative expression of carbohydrate epitopes MECA-79 in thin (*n* = 14) and normal (*n* = 18) endometrium. The bar represents optical density as a mean value ± standard deviation. *p < 0.05 compared to normal endometrium (**f**)

An intergroup comparison revealed a decreased expression of carbohydrate epitopes MECA-79 on the apical surface of the epithelial cells of thin endometrium, compared to normal (*р* = 0.033) (Fig. 2). Differences in glycoconjugates expression on the apical surface of the glandular cells were not found. An additional file shows this in more detail (see Table S2).

### Carbohydrate specificity of anti-Le^Y^ and MECA-79 MAbs

**Fig. 3:**
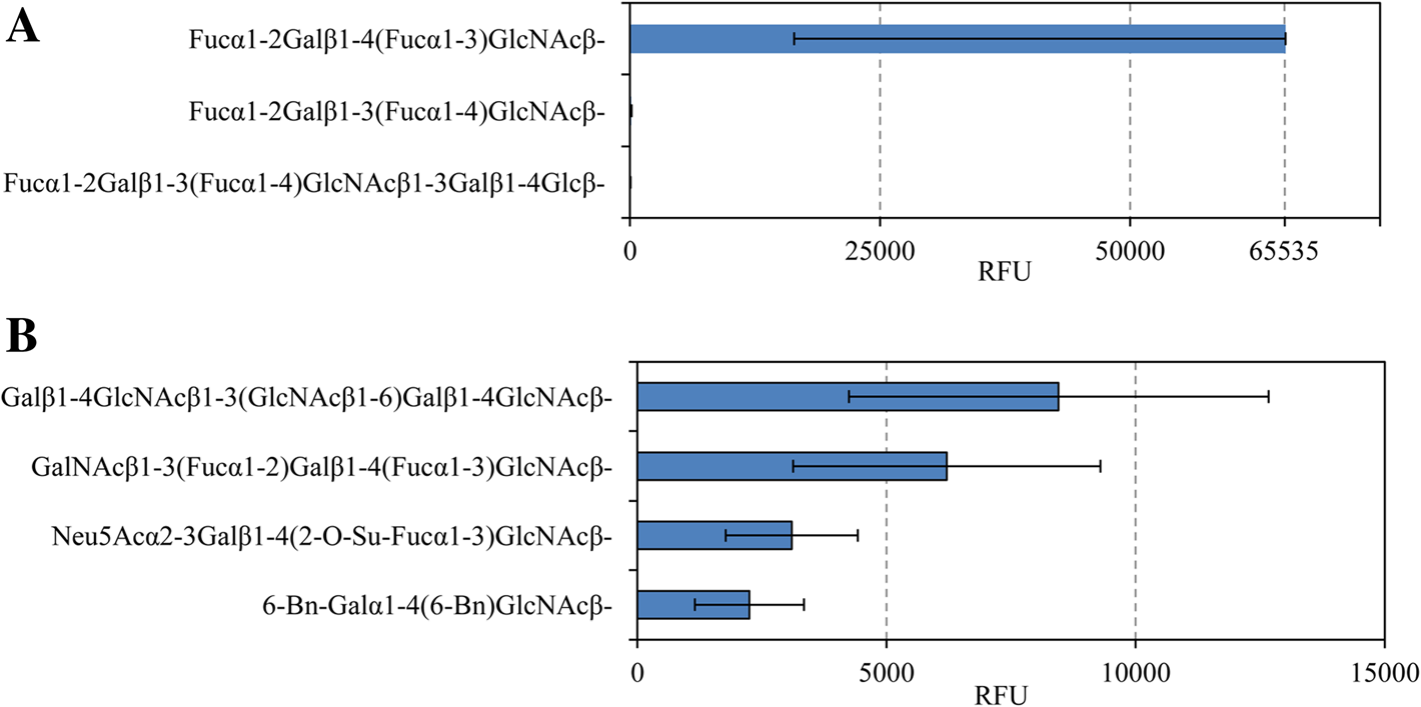
Top ligands for anti-Le^Y^ MAbs (**a**) and MECA-79 MAbs (**b**). For structures and short names of all ligands of the microarray chip see additional file (Table S1). Fucα1-2Galβ1-4(Fucα1-3) GlcNAcβ- (Le^Y^ #372). Fucα1-2Galβ1-3(Fucα1-4) GlcNAcβ- (Le^B^ #371). Fucα1-2Galβ1-3(Fucα1-4) GlcNAcβ1-3Galβ1-4Glcβ- (Le^B^Lac #496). Galβ1-4GlcNAcβ1-3(GlcNAcβ1-6) Galβ1-4GlcNAcβ (LN3′(GN6′)LN #489). GalNAcβ1-3(Fucα1-2) Galβ1-4(Fucα1-3) GlcNAcβ (ALe^Y^b #508). Neu5Acα2-3Galβ1-4(2-O-Su-Fucα1-3) GlcNAcβ (SiaLeX2′′′Su #431). 6-Bn-Galα1-4(6-Bn) GlcNAcβ (Bn2-aLN #126)

Microarray chip analysis of MAbs showed that anti-Le^Y^ MAbs exclusively bound to difucosylated oligosaccharide Le^Y^ (Fig. 3 a). The glycans, most actively binding to MECA-79 MAbs were those containing a type-2 unit LacNAc (Galβ1-4GlcNAcβ), and especially an oligolactosamine derivative Galβ1-4GlcNAcβ1-3(GlcNAcβ1-6) Galβ1-4GlcNAcβ. Lower but still significant binding was detected between MECA-79 MAbs and a Le^Y^ derivative GalNAcβ1-3(Fucα1-2)Galβ1-4(Fucα1-3)GlcNAcβ; a fucose-rich sulfated SialylLe^X^ residue Neu5Acα2-3Galβ1-4(2-O-Su-Fucα1-3)GlcNAcβ; and an α-lactosamine substituted derivative 6-Bn-Galα1-4(6-Bn) GlcNAcβ (where Bn is a benzyl residue) (Fig. 3b). Moreover, the 2nd and the 4th glycans from this list were not detected in humans before. I.e. MECA-79 MAbs specifically bind to LacNAc-containing oligosaccharides, but do not bind to mucin core 1 glycans of the microarray.

## Discussion

In this study, we explored the concept that the carbohydrate phenotype of endometrial structures may reflect a decreased receptivity of thin endometrium. We focused on glycans, basing on: 1) hormone-dependent change of the glycosylation pattern in the menstrual cycle [16, 27, 28]; 2) a specific endometrial glycosylation pattern associated with the blastocyst reception during the implantation window [13, 29–31]; 3) the crucial role of glycans in intercellular adhesion and tissue morphogenesis [32–35]. We chose to study the epithelial compartments of endometrium in patients with normal and thin endometrium since contact interactions occur on the apical surface of the endometrium epithelial cells, and the glycocalyx of the apical surface of glandular cells reflects their secretory activity and potential for intercellular interactions during trophoblast invasion [36].

Our study was the first to demonstrate that decreased receptivity in infertile female patients is associated with an altered glycan phenotype of thin endometrium. The investigation revealed reduced expression of the L-selectin ligand (MECA-79) in the glycocalyx of epithelial cells in thin endometrium. It should be noted that the glycan composition of MECA-79 epitope was intensively studied before. In particular, it was shown that desialylation of ClyCAM-1 enhanced binding of MECA-79 to ClyCAM-1. In contrast, binding of MECA-79 to ClyCAM-1 and CD34 significantly decreased when the sulfation of these ligands was reduced with chlorate, a metabolic inhibitor of sulfation. Defucosylation also significantly reduced the binding of MECA-79 with ClyCAM-1 [37]. Various authors denoted 6′-sulfated SialylLe^X^ (Siaα2-3(6-O-Su)Galβ1-4(Fucα1-3)GlcNAc on core 2 branched O-glycans [38], as well as sulfated extended core 1 mucin-type O-glycans Galβ1-4(6-O-Su)GlcNAcβ1-3GalNAc [23], as the MECA-79 epitope. The importance of a sulfated residue (6-O-Su)GlcNAc for binding to the epitope recognized by MECA-79 MAbs has been especially noted [39].

Our findings on MECA-79 MAbs specificity demonstrate the high binding activity of these antibodies to type-2 glycan unit LacNAc. Glycan Galβ1-4(6-O-Su)GlcNAcβ1-3GalNAc was not exposed on the microarray, but its sulfated form Galβ1-4(6-O-Su)GlcNAcβ (glycan № 203 in Table S1) was present, and we did not detect binding of MECA-79 to this glycan. Our data on the MECA-79 MAbs specificity do not contradict the literature, since our findings confirm the binding to LacNAc based glycans. However, (6-O-Su)GlcNAcβ residue is apparently not a unique factor for binding, but is recognized in complex with LacNAc, within a certain molecular recognition pattern of MECA-79 MAbs. Probably, a glycan epitope binding to MECA-79 is more complex than it has been shown before; this is quite explicable for MAbs obtained as a result of immunization with collagenase-dispersed BALB/c lymph node stroma, but not with one particular antigen.

MECA-79 is considered as one of the most relevant markers of endometrial receptivity [40]. A number of studies indicate that in a natural cycle, the expression of MECA-79 is significantly higher in fertile than in infertile patients [18]. Moreover, the absence of MECA-79 in the structures of endometrium in patients with infertility was shown to be associated with IVF failures [18]. Implantation and clinical pregnancy rates in patients with a higher MECA-79 expression were more than twice higher compared to patients with relatively low expression [41]. Our study allowed us to link lower MECA-79 expression with thin endometrium in patients with reproductive failures: embryo transfer in patients with endometrium less than 8 mm and decreased MECA-79 expression did not result in pregnancy, while 67% of patients with normal endometrium and higher MECA-79 expression succeeded to conceive. Therefore, assessment of MECA-79 expression may be used as a predictor of IVF failure and may help infertile patients with thin endometrium to avoid repeated useless treatment cycles.

The role of glycans in the pathophysiology of thin endometrium is also confirmed by the study of correlations between their expression levels in the main epithelial structures. The levels of expression of all studied glycans in two epithelial structures were comparable in patients of both groups, with a slight tendency towards higher expression in luminal epithelial cells. The luminal epithelium is the zone of primary contact with the trophectoderm, and the molecular “border” which the implanting embryo must overcome [13, 42]. The glands regulate uterine receptivity, luminal fluid homeostasis, blastocyst implantation, and embryo and placenta development at early stages [29, 36]. Both glandular and luminal epithelium determine the receptivity of the endometrium but perform different functions: regulation of implantation and reception of the embryo, respectively. It is known that during endometrial adenogenesis, differentiation and budding of glandular epithelium from luminal epithelium occurs, followed by invagination and extensive tubular coiling and branching through uterine stroma into the myometrium [36, 43, 44] Having differentiated from the luminal epithelium, the glandular compartment apparently retains the quantitative and, in general, the qualitative composition of the glycocalyx.

Worth noting is that a correlation between the contents of fucosylated glycoconjugates with type-2 unit stained with UEA-I, was found in the glycocalyx of luminal and glandular epithelial cells both in thin and normal endometrium. It is known that UEA-I specifically stains antigen H type 2 [Fucα1-2Galβ1-4GlcNAcβ-R], one of the five types of carbohydrate cores for biosynthesis of antigens of the AB0 system and related antigens of the Lewis system, including Le^Y^ [Fucα1-2Galβ1-4(Fucα1-3)GlcNAcβ-R] [45]. Galβ1-4GlcNAcβ is a precursor of both fucosylated antigens and is specifically detected by ECL lectin. In both study groups, a significant correlation between the contents of LacNac residues in the superficial and glandular epithelium was detected only after pretreatment of endometrial tissue with neuraminidase followed by staining with ECL (N + ECL); this indicates the interrelated expression of not terminal, but subterminal N-acetyllactosamine residues in both investigated structures. Noteworthy, in normal endometrium there is a tendency towards a higher expression of subterminal LacNAc residues in the surface epithelium, while in the thin endometrium, on the contrary, the expression of subterminal LacNAc residues it is higher in glandular epithelium. At the same time, the expression of subterminal N-acetyllactosamine residues in the epithelial compartments of patients of both groups is higher, than of other glycans; this demonstrates wide representation of glycans with a type-2 unit in the glycocalyx of the endometrial epithelial structures.

The terminal unsubstituted residues of LacNAc were found at the apical membranes of a few scattered glandular epithelial cells in normal endometrium, but could be seen more frequently in atrophic, weakly proliferating glandular epithelial cells [46]. We found no significant intergroup differences in the expression of both terminal and subterminal residues of N-acetyllactosamine, as well as of fucosylated glycans stained with UEA-I; however, similar correlations of the expression of the corresponding glycoconjugates in the studied epithelial compartments of thin and normal endometrium apparently reflect the universal patterns of expression of glycans’ functional residues, common for glycoconjugates of the endometrial glycocalyx, and may feature appropriate changes during the middle secretory phase, which do not affect endometrial receptivity.

In the study of α (1-2)-fucosylated glycans, special attention was paid to Le^Y^ glycan, one of the key molecules mediating cell junctions, which is expressed in the secretory phase of the cycle as a part of the carbohydrate chains of *α*v*β*3 integrin of the epithelial cells of the endometrium [47]. It was experimentally proved that Le^Y^ can function as a regulatory and signaling molecule, since its inhibition is associated with impaired expression of matrix metalloproteinase 9 (MMP-9), epidermal growth factor (EGF), epidermal growth factor receptor (EGFR), and with the blockade of the DAG/PKC signaling pathway, which regulates cell growth, division, and differentiation [48]. It was found that the expression of Le^Y^ and FUT4, an enzyme adding the Fucα1-3 residue to type-2 unit, correlates with a high reception of RL95-2 cells and activates the EGFR/MAPK signaling pathway [49]. Blocking of Le^Y^ glycan biosynthesis in RL-95-2 endometrial cells line in vitro leads to a decrease in their adhesion to JAR embryonic cells and negatively regulates the *α*v*β*3/FAK signaling pathway which is responsible for invasion and migration of invading cells [50]. This confirms the key role of Le^Y^ in intercellular contacts during implantation [45].

Our data showed that though overall Le^Y^ expression levels in normal and thin endometrium were almost similar, there was a certain correlation between the Le^Y^ levels in the two epithelial structures of thin endometrium, while in normal endometrium no correlation could be found. This phenomenon probably reflects the tissue homeostasis disorder, essential for morphogenesis of the endometrial functional layer and blastocyst acceptance, since Le^Y^ plays a key role in intercellular contacts during implantation [45]. The patterns of specificity of anti-Le^Y^ MAbs and UEA-I are similar, but not absolutely. As we have shown earlier, UEA-I has a broader specificity and recognizes fucosylated LacNAc structures, including H type 2 and Le^Y^ antigens and some other LacNAc-containing glycans [51]. Apparently, thin endometrium has specific Le^Y^ glycan expression which affects the endometrium receptivity, while expression of glycans containing type-2 unit is crucial for endometrial tissue, since LacNAc and its derivatives are the basis for biosynthesis of branched glycans abundantly present in the endometrium [52]. A pattern similar to Le^Y^ was observed for mannose-rich glycans (staining with Con A) in the epithelial compartments of thin endometrium. A review of available literature provided evidence of an altered pattern of Con A binding in atrophic and hyperplastic postmenopausal endometrium [53], and in neoplastic endometrium with poor histological differentiation [54]. We have not found such correlations in normal endometrium. We assume that with the differentiation of luminal epithelium into the glandular epithelium and with the emergence of new functions in the normal endometrium with high receptivity, the correlation between the expression of certain glycans in the two epithelial structures vanish, reflecting adequate differentiation of epithelial cells. In thin endometrium the processes of differentiation of the glandular epithelium are probably impaired; this may negatively affect the maturity and functional activity of the cells.

## Conclusion

Therefore, our preliminary study revealed specific expression of mannose-rich glycans and LacNAc-containing oligosaccharides (including Le^Y^ and glycans containing LacNAc type-2 unit, which have been identified as potential ligands to MECA-79 MAbs) in the epithelial compartments of thin endometrium involved in the interactions with the blastocyst during the implantation window. These changes may be essential for morphogenesis of the endometrial functional layer, resulting in the tissue deficiency and incapability to support the development of a haemochorial interface, and this, in turn, may affect the blastocyst implantation. We believe that alteration in the endometrial glycosylation pattern, namely the glycocalyx composition in the epithelial compartments, may contribute to the pathophysiology of thin endometrium and explain its low receptivity. Undoubtedly, a more profound study of a specific glycosylation pattern may provide insight into the pathophysiology of thin endometrium.

## Supplementary Information

**Additional file 1: Table S1.** The list of glycans. This Table represents all glycans (oligo- and polysaccharides) printed on microarrays used in this work.

**Additional file 2: Table S2.** Glycoconjugates contents on the apical surface of the luminal and glandular epithelial cells in patients with “thin” endometrium (group 1) and patients with normal endometrium (group 2).

## Data Availability

The datasets used and/or analyzed during the current study are available from the corresponding author on reasonable request.

## Abbreviations

AMH: Anti Mullerian hormone
ART: Assisted reproductive technologies
BMI: Body mass index
Con A: Сoncanavalin A from *Canavalia ensiformis*
EGF: Epidermal growth factor
EGFR: Epidermal growth factor receptor
ECL: *Erythrina cristagalli* lectin
ET: Embryo transfer
FSH: Follicle-stimulating hormone
FUT4: Fucosyltransferase 4
GlyCAM-1: Glycosylation-dependent cell adhesion molecule-1
GT: Gonadotropins
GnRH-ant: Gonadotropin-releasing hormone antagonists
IHC: Immunohistochemical
ICSI: Intracytoplasmic sperm injection
IVF: In vitro fertilization
LacNAc: N-acetyllactosamine
LH: Luteinizing hormone
Le^Y^: Lewis^Y^
MAL-II: *Maackia amurensis* lectin-II
MMP-9: Matrix metalloproteinase 9
MAbs: Monoclonal antibodies
MAdCAM-1: Mucosal addressin cell adhesion molecule-1
N: Neuraminidase
PNAd: Peripheral lymph node addressin
rFSH: Recombinant follicle-stimulating hormone
RFU: Relative fluorescence units
SNA: *Sambucus nigra* lectin
TVP: Transvaginal puncture
UEA-I: *Ulex europaeus* agglutinin-I
VVL: *Vicia villosa* lectin
