# Supplementary material for: Epithelial apical glycosylation changes associated with thin endometrium in women with infertility - a pilot observational study
Source: Reprod Biol Endocrinol. 2021 May 15;19:73. doi: 10.1186/s12958-021-00750-z (PMC8122553; doi:10.1186/s12958-021-00750-z)
Supplement: Supplementary file 1 — Additional file 1: Table S1. The list of glycans. This Table represents all glycans (oligo- and polysaccharides) printed on microarrays used in this work. [file 12958_2021_750_MOESM1_ESM.docx]

Table S1. The list of glycans. This Table represents all glycans (oligo- and polysaccharides) printed on microarrays used in this work

| Nr | Structure | Short Name |
| --- | --- | --- |
| 001 | Fucα-sp3 | aF |
| 002 | Galα-sp3 | aA |
| 003 | Galβ-sp3 | bA |
| 004 | GalNAcα1-OSer | TnSer |
| 005 | GalNAcα-sp3 | Tn |
| 006 | GalNAcβ-sp3 | bAN |
| 007 | Glcα-sp3 | aG |
| 009 | Glcβ-sp3 | bG |
| 010 | GlcNAcβ-sp3 | GN |
| 011 | GlcNAcβ-sp2 | GN-C2 |
| 013 | GlcNAcβ-sp8 | GN-PEG |
| 014 | GlcN(Gc)β-sp4 | bGN(Gc) |
| 016 | Manα-sp3 | aM |
| 017 | Manα-sp4 | aM-Gly |
| 018 | Manβ-sp4 | bM |
| 019 | ManNAcβ-sp4 | bMN |
| 020 | Rhaα-sp3 | aR |
| 021 | Galβ-sp4 | bA-Gly |
| 022 | GlcNAcβ-sp4 | GN-Gly |
| 023 | GalNAcβ-sp4 | bAN-Gly |
| 025 | GalNAcβ-sp10 | bAN-PEG2 |
| 026 | Rhaβ-sp4 | bR |
| 027 | 3,6-O-Me_2_-Glcβ-sp3 | 3,6-Me2Glc |
| 028 | Xylβ-sp4 | bXyl |
| 029 | Fucβ-sp4 | bF |
| 030 | Glcβ-sp4 | bG-Gly |
| 031 | L-Araα-sp4 | aAra |
| 032 | GalN(Gc)β-sp3 | bANGc |
| 037 | 3-O-Su-Galβ-sp3 | bA3Su |
| 038 | 3-O-Su-GalNAcβ-sp3 | bAN3Su |
| 041 | 6-O-Su-GalNAcα-sp3 | aAN6Su |
| 043 | 6-O-Su-GlcNAcβ-sp3 | GN6Su |
| 044 | GlcAα-sp3 | aGU |
| 045 | GlcAβ-sp3 | bGU |
| 046 | 6-H_2_PO_3_Glcβ-sp4 | G6P |
| 047 | 6-H_2_PO_3_Manα-sp3 | M6P |
| 048 | Neu5Acα-sp3 | Sia |
| 049 | Neu5Acα-sp9 | Sia-Bn |
| 050 | Neu5Acβ-sp3 | bSia |
| 051 | Neu5Acβ-sp9 | bSia-Bn |
| 052 | Neu5Gcα-sp3 | aNeu5Gc |
| 053 | Neu5Gcβ-sp3 | bSia5Gc |
| 054 | 9-NAc-Neu5Acα-sp3 | 9NAcSia |
| 055 | 3-O-Su-GlcNAcβ-sp3 | GN3Su |
| 057 | D-Ribβ -sp4 | bRib |
| 058 | Fucβ-sp3 | bF |
| 059 | αKdo-5-phosphate-sp11 | A173 |
| 060 | 6-O-Su-Galβ-sp3 | bA6Su |
| 061 | 3-O-Su-GalNAcα-sp3 | Tn3Su |
| 062 | GlcAβ-sp2 | bGN-C2 |
| 063 | 4-O-Su-GlcNAcβ-sp2 | GN4Su |
| 064 | 4-O-Su-Galβ-sp3 | A4Su |
| 065 | 4-O-Su-GalNAcα-sp3 | Tn4Su |
| 066 | 4-O-Su-GalNAcβ-sp3 | bAN4Su |
| 071 | Fucα1-2Galβ-sp3 | Hdi |
| 072 | Fucα1-3GlcNAcβ-sp3 | Fa3GN |
| 073 | Fucα1-4GlcNAcβ-sp3 | Le |
| 074 | Fucβ1-3GlcNAcβ-sp3 | Fb3GN |
| 075 | Galα1-2Galβ-sp3 | Aa2A |
| 076 | Galα1-3Galβ-sp3 | Bdi |
| 077 | Galα1-3GalNAcβ-sp3 | Tab |
| 078 | Galα1-3GalNAcα-sp3 | Taa |
| 080 | Galα1-3GlcNAcβ-sp3 | Aa3GN |
| 081 | Galα1-4GlcNAcβ-sp3 | aLN |
| 082 | Galα1-4GlcNAcβ-sp8 | aLN-PEG |
| 083 | Galα1-6Glcβ-sp4 | Aa6G |
| 084 | Galβ1-2Galβ-sp3 | Ab2A |
| 085 | Galβ1-3GlcNAcβ-sp3 | LeC |
| 086 | Galβ1-3GlcNAcβ-sp2 | LeC-C2 |
| 087 | Galβ1-3Galβ-sp3 | Ab3A |
| 089 | Galβ1-3GalNAcα-sp3 | TF |
| 092 | Galβ1-4Glcβ-sp2 | Lac-C2 |
| 093 | Galβ1-4Glcβ-sp4 | Lac-Gly |
| 094 | Galβ1-4Galβ-sp4 | Ab4A |
| 096 | Galβ1-4GlcNAcβ-sp2 | LN-C2 |
| 097 | Galβ1-4GlcNAcβ-sp3 | LN |
| 098 | Galβ1-4GlcNAcβ−sp5 | LN-C8 |
| 099 | Galβ1-4GlcNAcβ-sp8 | LN-PEG |
| 100 | Galβ1-6Galβ-sp4 | Ab6A |
| 101 | GalNAcα1-3GalNAcβ-sp3 | Fs-2 |
| 102 | GalNAcα1-3Galβ-sp3 | Adi |
| 103 | GalNAcα1-3GalNAcα-sp3 | core5 |
| 104 | GalNAcβ1-3Galβ-sp3 | ANb3A |
| 105 | GalNAcβ1-3GalNAcβ-sp3 | para-Fs |
| 106 | GalNAcβ1-4GlcNAcβ-sp3 | LacdiNAc |
| 107 | GalNAcβ1-4GlcNAcβ-sp2 | LacdiNAc-C2 |
| 110 | Glcα1-4Glcβ-sp3 | Malt2 |
| 111 | Glcβ1-4Glcβ-sp4 | cello |
| 112 | Glcβ1-6Glcβ-sp4 | gent |
| 113 | GlcNAcβ1-3GalNAcα-sp3 | core3 |
| 114 | GlcNAcβ1-3Manβ-sp4 | GN3M |
| 115 | GlcNAcβ1-4GlcNAcβ-Asn | Ch2-Asn |
| 116 | GlcNAcβ1-4GlcNAcβ-sp3 | Ch2 |
| 117 | GlcNAcβ1-4GlcNAcβ-sp4 | Ch2-Gly |
| 118 | GlcNAcβ1-6GalNAcα-sp3 | core6 |
| 119 | Manα1-2Manβ-sp4 | Ma2Mb |
| 120 | Manα1-3Manβ-sp4 | Ma3M |
| 121 | Manα1-4Manβ-sp4 | Ma4M |
| 122 | Manα1-6Manβ-sp4 | Ma6M |
| 123 | Manβ1-4GlcNAcβ-sp4 | Mb4GN |
| 125 | 6-Bn-Galβ1-4GlcNAcβ-sp2 | 6'Bn-LN |
| 126 | 6-Bn-Galα1-4(6-Bn)GlcNAcβ-sp3 | Bn2-aLN |
| 127 | Galβ1-4Glcβ-sp4-Phe | Lac-Phe |
| 128 | Galβ1-4Glcβ-sp4-Trp | Lac-Trp |
| 129 | Galβ1-3(6-O-Bn)GlcNAcβ-sp3 | 6BnLeC |
| 130 | (6-O-Bn-Galβ1)-3GlcNAcβ-sp2 | 6'BnLeC |
| 131 | (6-O-Bn-Galβ1)-3(6-O-Bn)GlcNAcβ-sp3 | Bn2LeC |
| 132 | Galβ1-3GalNAcα-sp5 | TF-С8 |
| 133 | Galβ1-4Glcβ-sp4-Ala | Lac-Ala |
| 134 | Galβ1-4Glcβ-sp4-Arg | Lac-Arg |
| 135 | Galβ1-4Glcβ-sp4-Asn | Lac-Asn |
| 136 | Galβ1-4Glcβ-sp4-Ile | Lac-Ile |
| 137 | Galβ1-4Glcβ-sp4-Nle | Lac-Nle |
| 138 | Galβ1-4Glcβ-sp4-Val | Lac-Val |
| 139 | Galβ1-4GlcNAcα-sp3 | LNa |
| 140 | Galα1-3GalNAc(fur)β-sp3 | Tab(f) |
| 142 | GlcNAcα1-3GalNAcβ-sp3 | GNa3AN |
| 143 | Fucα1-2(3-O-Su)Galβ-sp3 | Hdi3Su |
| 144 | Galβ1-3(6-O-Su)GlcNAcβ-sp2 | LeC6Su-C2 |
| 145 | Galβ1-3(6-O-Su)GlcNAcβ-sp3 | LeC6Su |
| 146 | Galβ1-4(6-O-Su)Glcβ-sp2 | Lac6Su |
| 149 | GlсNAcβ1-4(6-O-Su)GlcNAcβ-sp2 | Ch2-6Su |
| 150 | 3-O-Su-Galβ1-3GalNAcα-sp3 | TF3'Su |
| 151 | 6-O-Su-Galβ1-3GalNAcα-sp3 | TF6'Su |
| 152 | 3-O-Su-Galβ1-4Glcβ-sp2 | Lac3'Su |
| 153 | 6-O-Su-Galβ1-4Glcβ-sp2 | Lac6'Su |
| 154 | 3-O-Su-Galβ1-3GlcNAcβ-sp3 | LeC3'Su |
| 156 | 3-O-Su-Galβ1-4GlcNAcβ-sp2 | LN3'Su-C2 |
| 158 | 4-O-Su-Galβ1-4GlcNAcβ-sp2 | LN4'Su-C2 |
| 159 | 4-O-Su-Galβ1-4GlcNAcβ-sp3 | LN4'Su |
| 160 | 6-O-Su-Galβ1-3GlcNAcβ-sp2 | LeC6'Su-C2 |
| 161 | 6-O-Su-Galβ1-3GlcNAcβ-sp3 | LeC6'Su |
| 162 | 6-O-Su-Galβ1-4GlcNAcβ-sp2 | LN6'Su-C2 |
| 164 | GlcAβ1-3GlcNAcβ-sp3 | GUb3GN |
| 165 | GlcAβ1-3Galβ-sp3 | GUb3A |
| 166 | GlcAβ1-6Galβ-sp3 | GUb6A |
| 167 | GlcNAcβ1-4-[HOOC(CH_3_)CH]-3-O-GlcNAcβ-sp4 | GN-Mur |
| 168 | GlcNAcβ1-4Mur-L-Ala-D-i-Gln-Lys | GMDPLys |
| 169 | Neu5Acα2-3Galβ-sp3 | GM4 |
| 170 | Neu5Acα2-6Galβ-sp3 | Sia6A |
| 171 | Neu5Acα2-3GalNAcα-sp3 | 3-SiaTn |
| 172 | Neu5Acα2-6GalNAcα-sp3 | SiaTn |
| 173 | Neu5Acβ2-6GalNAcα-sp3 | bSiaTn |
| 174 | Neu5Gcα2-6GalNAcα-sp3 | Neu5GcTn |
| 175 | Neu5Gcβ2-6GalNAcα-sp3 | bNeu5GcTn |
| 176 | 3-O-Su-Galβ1-4(6-O-Su)Glcβ-sp2 | Lac3',6Su2 |
| 177 | 3-O-Su-Galβ1-4(6-O-Su)GlcNAcβ-sp3 | LN3'6Su2 |
| 178 | 6-O-Su-Galβ1-4(6-O-Su)Glcβ-sp2 | Lac6,6'Su2 |
| 179 | 6-O-Su-Galβ1-3(6-O-Su)GlcNAcβ-sp2 | LeC6,6'Su2 |
| 180 | 6-O-Su-Galβ1-4(6-O-Su)GlcNAcβ-sp2 | LN66'Su2 |
| 181 | 3,4-O-Su_2_-Galβ1-4GlcNAcβ-sp3 | LN3'4'Su2 |
| 182 | 3,6-O-Su_2_-Galβ1-4GlcNAcβ-sp2 | LN3'6'Su2 |
| 183 | 4,6-O-Su_2_-Galβ1-4GlcNAcβ-sp2 | LN4'6'Su2-C2 |
| 184 | 4,6-O-Su_2_-Galβ1-4GlcNAcβ-sp3 | LN4'6'Su2 |
| 186 | Neu5Acα2-8Neu5Acα2-sp3 | (Sia)2 |
| 187 | Neu5Acα2-8Neu5Acα2-sp9 | (Sia)2Bn |
| 188 | Neu5Acα2-8Neu5Acβ-sp9 | (Sia)2-bBn |
| 189 | 3,6-O-Su_2_-Galβ1-4(6-O-Su)GlcNAcβ-sp2 | LN3'66'Su3 |
| 190 | Galβ1-4-(6-P)GlcNAcβ-sp2 | LN6P |
| 191 | 6-P-Galβ1-4GlcNAcβ-sp2 | LN6'P |
| 192 | GalNAcβ1-4(6-O-Su)GlcNAcβ-sp3 | LacdiNAc6Su |
| 193 | 3-O-Su-GalNAcβ1-4GlcNAcβ-sp3 | LacdiNAc3'Su |
| 194 | 6-O-Su-GalNAcβ1-4GlcNAcβ-sp3 | LacdiNAc6'Su |
| 195 | 6-O-Su-GalNAcβ1-4(3-O-Ac)GlcNAcβ-sp3 | 3Ac-LacdiNAc6'Su |
| 196 | 3-O-Su-GalNAcβ1-4(3-O-Su)GlcNAcβ-sp3 | LacdiNAc3,3'Su2 |
| 197 | 3,6-O-Su_2_-GalNAcβ1-4GlcNAcβ-sp3 | LacdiNAc3',6'Su2 |
| 198 | 4,6-O-Su_2_-GalNAcβ1-4GlcNAcβ-sp3 | LacdiNAc4',6'Su2 |
| 199 | 4,6-O-Su_2_-GalNAcβ1-4-(3-O-Ac)GlcNAcβ-sp3 | 3Ac-LacdiNAc4',6'Su2 |
| 200 | 4-O-Su-GalNAcβ1-4GlcNAcβ-sp3 | LacdiNAc4'Su |
| 201 | 3,4-O-Su_2_-GalNAcβ1-4GlcNAcβ-sp3 | LacdiNAc3',4'Su2 |
| 202 | 6-O-Su-GalNAcβ1-4(6-O-Su)GlcNAcβ-sp3 | LacdiNAc6,6'Su2 |
| 203 | Galβ1-4(6-O-Su)GlcNAcβ-sp2 | LN6Su |
| 204 | 4-O-Su-GalNAcβ1-4GlcNAcβ-sp2 | LacdiNAc4'Su-C2 |
| 205 | Neu5Acα2-6GalNAcβ-sp3 | 6SiaANb |
| 206 | Neu5Gcα2-3Galβ-sp3 | Neu5Gc3A |
| 207 | Neu5Acβ2-6GalNAcβ-sp3 | bSiaANb |
| 208 | Galβ1-3GlcNAcβ-sp4 | LeC-Gly |
| 209 | αKdo-(2→4)-αKdo-sp11 | A58 |
| 210 | L,D-Hep-(1→5)-αKdo-4-phosphate-sp11 | JN |
| 211 | αKdo-(2→8)-αKdo-sp11 | A78 |
| 212 | D-glycero-α-D-talo-octulosonic acid (Ko)-(2→4)-αKdo-sp11 | NW61S |
| 213 | Neu5Acβ2-6Galβ-sp3 | 6bSiaA |
| 214 | ΔGlcAβ1-3Galβ-sp3 | deltaGUb3A |
| 215 | Fucα1-2Galβ1-3GlcNAcβ-sp3 | LeD |
| 216 | Fucα1-2Galβ1-4GlcNAcβ-sp3 | Htype2 |
| 217 | Fucα1-2Galβ1-3GalNAcα-sp3 | Htype3 |
| 219 | Fucα1-2Galβ1-4Glcβ-sp4 | Htype6 |
| 220 | Galα1-3Galβ1-4Glcβ-sp2 | Aa3'Lac-C2 |
| 221 | Galα1-3Galβ1-4Glcβ-sp4 | Aa3'Lac-Gly |
| 222 | Galα1-3Galβ1-4GlcNAcβ-sp3 | Galili3 |
| 223 | Galα1-4Galβ1-4Glcβ-sp2 | Pk-C2 |
| 225 | Galα1-4Galβ1-4GlcNAcβ-sp2 | P1 |
| 226 | Galα1-3(Fucα1-2)Galβ-sp3 | Btri |
| 227 | Galα1-3(Fucα1-2)Galβ-sp5 | Btri-C8 |
| 228 | Galβ1-2Galα1-3GlcNAcβ-sp3 | Ab2Aa3GN |
| 229 | Galβ1-3Galβ1-4GlcNAcβ-sp4 | Ab3'LN-Gly |
| 231 | Galβ1-4GlcNAcβ1-3GalNAcα−sp3 | LN3Tn |
| 232 | Galβ1-4GlcNAcβ1-6GalNAcα−sp3 | LN6Tn |
| 233 | Galβ1-3(Fucα1-4)GlcNAcβ-sp3 | LeA |
| 234 | Galβ1-4(Fucα1-3)GlcNAcβ-sp3 | LeX |
| 235 | GalNAcα1-3(Fucα1-2)Galβ-sp3 | Atri |
| 236 | GalNAcα1-3(Fucα1-2)Galβ-sp5 | Atri-C8 |
| 237 | GalNHα1-3(Fucα1-2)Galβ-OCH_2_CH_2_CH_2_NHAc | ABtri |
| 239 | GalNAcβ1-3(Fucα1-2)Galβ-sp3 | Fa2(ANb3)A |
| 240 | (Glcα1-4)_3_β-sp4 | (Ga4)3b |
| 241 | (Glcα1-6)_3_β-sp4 | (Ga6)3b |
| 242 | GlcNAcα1-3Galβ1-4GlcNAcβ-sp2 | GNa3'LN-C2 |
| 243 | GlcNAcα1-3Galβ1-4GlcNAcβ-sp3 | GNa3'LN |
| 245 | GlcNAcα1-6Galβ1-4GlcNAcβ-sp2 | GNa6'LN |
| 246 | GlcNAcβ1-2Galβ1-3GalNAcα-sp3 | GN2'TF |
| 247 | GlcNAcβ1-3Galβ1-3GalNAcα-sp3 | GN3'TF |
| 248 | GlcNAcβ1-3Galβ1-4Glcβ-sp2 | GN3'Lac |
| 249 | GlcNAcβ1-3Galβ1-4GlcNAcβ-sp2 | GN3'LN-C2 |
| 250 | GlcNAcβ1-3Galβ1-4GlcNAcβ-sp3 | GN3'LN |
| 251 | GlcNAcβ1-4Galβ1-4GlcNAcβ-sp2 | GN4'LN |
| 252 | GlcNAcβ1-4GlcNAcβ1-4GlcNAcβ-sp4 | Ch3 |
| 253 | GlcNAcβ1-6Galβ1-4GlcNAcβ-sp2 | GN6'LN |
| 254 | GlcNAcβ1-6(Galβ1-3)GalNAcα-sp3 | core2 |
| 255 | GlcNAcβ1-6(GlcNAcβ1-3)GalNAcα-sp3 | core4 |
| 256 | GlcNAcβ1-6(GlcNAcβ1-4)GalNAcα-sp3 | GN2-4,6Tn |
| 258 | Manα1-6(Manα1-3)Manβ-sp4 | (Ma)3b |
| 259 | Galβ1-4(Galβ1-3)GlcNAcβ-sp3 | (Ab)2-3,4GN |
| 260 | Galβ1-3(Fucβ1-4)GlcNAcβ-sp3 | bLeA |
| 261 | Galβ1-4(Fucβ1-3)GlcNAcβ-sp3 | bLeX |
| 262 | Galβ1-3GalNAcβ1-3Galβ-sp4 | Tbb-A |
| 263 | (GalNAcβ-PEG_2_)_3_-β-DD | ANb-cluster |
| 264 | Galβ1-4Galβ1-4GlcNAcβ-sp3 | Ab4'LN |
| 266 | Galα1-4Galβ1-4GlcNAcβ-sp3 | P1 |
| 267 | GlcNAcβ1-3Galβ1-3GlcNAcβ-sp3 | GlcNAc3'Le^c^ |
| 268 | GlсNAcβ1-4(Fucα1-6)GlcNAcβ-sp3 | Fa6Ch2 |
| 269 | Galβ1-3Galβ1-4Glcβ-sp4 | Gal3'Lac |
| 270 | Galβ1-4Galβ1-4Glcβ-sp4 | Gal4'Lac |
| 271 | Galβ1-6Galβ1-4Glcβ-sp4 | Gal6'Lac |
| 272 | Neu5Acα2-3Galβ1-4Glcβ-sp4-Cit | 3'SL-Cit |
| 273 | Fucβ1-2Galβ1-4GlcNAcβ-sp3 | Fb2LN |
| 274 | GalNAcα1-3Galβ1-4GlcNAcβ-sp3 | ANa3'LN |
| 275 | GalNAcβ1-3Galβ1-4GlcNAcβ-sp3 | ANb3'LN |
| 276 | GlcNAcβ1-4Galβ1-4GlcNAcβ-sp3 | GN4'LN-C3 |
| 277 | GalN(Gc)α1-3(Fucα1-2)Galβ-sp3 | NGcAtri |
| 278 | GalNAcα1-3GalNAcβ1-3Galβ-sp3 | Fs-3 |
| 279 | Galβ1-3GlcNAcα1-3GalNAcα-sp3 | LeCa3Tn |
| 280 | Galβ1-3GlcNAcβ1-3GalNAcα-sp3 | LeC3Tn |
| 281 | Fucα1-2Galβ1-3GalNAcβ-sp3 | Htype4 |
| 288 | 3-O-Su-Galβ1-4(Fucα1-3)GlcNAcβ-sp3 | 3'SuLeX |
| 289 | Neu5Acα2-6(Galβ1-3)GalNAcα-sp3 | 6SiaTF |
| 290 | Neu5Acα2-6(Galα1-3)GalNAcα-sp3 | A3a(Sia)Tn |
| 291 | Neu5Acβ2-6(Galβ1-3)GalNAcα-sp3 | b6SiaTF |
| 292 | Neu5Acα2-3Galβ1-3GalNAcα-sp3 | Sia3'TF |
| 293 | Neu5Acα2-3Galβ1-4Glcβ-sp3 | 3'SL |
| 294 | Neu5Acα2-3Galβ1-4Glcβ-sp4 | 3'SL-Gly |
| 295 | Neu5Acα2-6Galβ1-4Glcβ-sp2 | 6'SL-C2 |
| 296 | Neu5Acα2-6Galβ1-4Glcβ-sp4 | 6'SL-Gly |
| 297 | Neu5Acβ2-6Galβ1-4Glcβ-sp2 | b6'SL |
| 298 | Neu5Acα2-3Galβ1-4GlcNAcβ-sp3 | 3'SLN |
| 299 | Neu5Acα2-3Galβ1-3GlcNAcβ-sp3 | 3'SiaLeC |
| 300 | Neu5Acα2-6Galβ1-4GlcNAcβ-sp3 | 6'SLN |
| 302 | Neu5Acβ2-6Galβ1-4GlcNAcβ-sp3 | b6'SLN |
| 303 | Neu5Gcα2-3Galβ1-4GlcNAcβ-sp3 | 3'SLN(Gc) |
| 304 | Neu5Gcα2-6Galβ1-4GlcNAcβ-sp3 | 6'SLN(Gc) |
| 305 | Neu5Gcβ2-6Galβ1-4GlcNAcβ-sp3 | b6'SLN(Gc) |
| 306 | 9-NAc-Neu5Acα2-6Galβ1-4GlcNAcβ-sp3 | 9NAc-6'SLN |
| 307 | KDNα2-3Galβ1-3GlcNAcβ-sp2 | KDN3'LeC |
| 308 | KDNα2-3Galβ1-4GlcNAcβ-sp2 | KDN3'LN |
| 309 | Neu5Acα2-6(Neu5Acα2-3)GalNAcα-sp3 | Sia2-3,6Tn |
| 310 | Neu5Acα2-3Galβ1-4GlcNAcβ-O(CH_2_)_3_NH-amide-Neu5Acα2-3Galβ1-4GlcNAcβ-sp3 | (3'SLN)2 |
| 315 | Neu5Acα2-3Galβ1-4-(6-O-Su)GlcNAcβ-sp3 | 3'SLN6Su |
| 316 | Fucα1-2(6-O-Su)Galβ1-4GlcNAcβ-sp3 | 6'SuHtype2 |
| 318 | Neu5Acα2-6Galβ1-4(6-O-Su)GlcNAcβ-sp3 | 6'SLN6Su |
| 319 | Neu5Acα2-3(6-O-Su)Galβ1-4GlcNAcβ-sp3 | 3'SLN6'Su |
| 320 | 4-O-Su-Neu5Acα2-3(6-O-Su)Galβ1-4GlcNAcβ-sp3 | 3'SLN6',4''Su2 |
| 321 | (Neu5Acα2-8)_3_-sp3 | (Sia)3 |
| 322 | (Neu5Acα2-8)_3_β-sp3 | (Sia)3b |
| 323 | Neu5Acα2-6Galβ1-3GlcNAcβ-sp3 | 6'SiaLeC |
| 324 | Neu5Acα2-6Galβ1-3(6-O-Su)GlcNAcβ-sp3 | 6'SiaLeC6Su |
| 325 | Neu5Acα2-3Galβ1-4Glcβ-sp4-Ala | 3'SL-Ala |
| 326 | Neu5Acα2-3Galβ1-4Glcβ-sp4-Ile | 3'SL-Ile |
| 327 | Neu5Acα2-3Galβ1-4Glcβ-sp4-Nle | 3'SL-Nle |
| 328 | Neu5Acα2-3Galβ1-4Glcβ-sp4-Phe | 3'SL-Phe |
| 329 | Neu5Acα2-3Galβ1-4Glcβ-sp4-Trp | 3'SL-Trp |
| 330 | Neu5Acα2-3Galβ1-4Glcβ-sp4-Val | 3'SL-Val |
| 331 | Neu5Gcα2-3Galβ1-3GlcNAcβ-sp3 | 3'SiaLeC(Gc) |
| 332 | Neu5Acα2-3Galβ1-4Glcβ-sp4-Asn | 3’SL-Asn |
| 333 | Neu5Gcα2-3Galβ1-3(6-O-Su)GlcNAcβ-sp3 | 3'SiaLeC(Gc)6Su |
| 334 | Neu5Gcα2-3Galβ1-4(6-O-Su)GlcNAcβ-sp3 | 3'SLN(Gc)6Su |
| 335 | Neu5Acα2-3Galβ1-3(6-O-Su)GlcNAcβ-sp3 | 3'SiaLeC6Su |
| 336 | αKdo-(2→8)-αKdo-(2→4)-αKdo-sp11 | A128 |
| 337 | GalNAcα1-4Galβ1-4GlcNAcβ-sp3 | ANa4'LN |
| 338 | Neu5Acα2-6Galβ1-3GalNAcα-sp3 | 6'SiaTF |
| 339 | Neu5Acβ2-6Galβ1-3GalNAcα-sp3 | 6'bSiaTF |
| 340 | Galα1-3(Neu5Acβ2-6)GalNAcβ-sp3 | b6SiaTab |
| 341 | Neu5Acα2-3-(6-O-Su)Galβ1-4GlcNAcβ-sp2 | 6'Su3'SLN |
| 342 | Neu5Acα2-3(6-O-Su)Galβ1-3GalNAcα-sp3 | 6'Su3'SiaTF |
| 343 | Neu5Gcα2-3Galβ1-4Glcβ-sp2 | 3'SL(Gc) |
| 344 | Neu5Acβ2-3Galβ1-4GlcNAcβ-sp3 | b3’SLN |
| 345 | Neu5Acα2-3Galβ1-3(6-O-Su)GalNAcα-sp3 | 6Su3'SiaTF |
| 346 | Neu5Acα2-3-(6-O-Su)Galβ1-4(6-O-Su)GlcNAcβ-sp3 | 6,6'Su2-3'SLN |
| 359 | Galα1-3(Fucα1-2)Galβ1-3GlcNAcβ-sp3 | Btype1 |
| 360 | Galα1-3(Fucα1-2)Galβ1-4GlcNAcβ-sp3 | Btype2 |
| 361 | Galα1-3(Fucα1-2)Galβ1-4GlcNAcβ-sp2 | Btype2-C2 |
| 362 | Galα1-3(Fucα1-2)Galβ1-3GalNAcα-sp3 | Btype3 |
| 363 | Galα1-3(Fucα1-2)Galβ1-3GalNAcβ-sp3 | Btype4 |
| 364 | Galα1-3Galβ1-4(Fucα1-3)GlcNAcβ-sp3 | aGalLeX |
| 365 | Galα1-4(Fucα1-2)Galβ1-4GlcNAcβ-sp3 | Aa4'(Fa2')LN |
| 366 | GalNAcα1-3(Fucα1-2)Galβ1-3GlcNAcβ-sp3 | Atype1 |
| 368 | GalNAcα1-3(Fucα1-2)Galβ1-4GlcNAcβ-sp3 | Atype2 |
| 369 | GalNAcα1-4(Fucα1-2)Galβ1-4GlcNAcβ-sp3 | ANa4'(Fa2')LN |
| 370 | GalNAcβ1-3(Fucα1-2)Galβ1-4GlcNAcβ-sp3 | ANb3'(Fa2')LN |
| 371 | Fucα1-2Galβ1-3(Fucα1-4)GlcNAcβ-sp3 | LeB |
| 372 | Fucα1-2Galβ1-4(Fucα1-3)GlcNAcβ-sp3 | LeY |
| 373 | Galα1-3Galβ1-4GlcNAcβ1-3Galβ-sp3 | Galili4 |
| 374 | Galα1-3(Galα1-4)Galβ1-4GlcNAcβ-sp3 | Aa2-3',4'LN |
| 376 | Galβ1-3GlcNAcβ1-3Galβ1-4Glcβ-sp4 | LNT |
| 377 | Galβ1-3GlcNAcβ1-3Galβ1-3GlcNAcβ-sp2 | LeCb3'LeC |
| 378 | Galβ1-3GlcNAcα1-3Galβ1-4GlcNAcβ-sp3 | LeCa3'LN |
| 379 | Galβ1-3GlcNAcβ1-3Galβ1-4GlcNAcβ-sp3 | LeC3'LN |
| 380 | Galβ1-3GlcNAcα1-6Galβ1-4GlcNAcβ-sp2 | LeCa6'LN |
| 381 | Galβ1-3GlcNAcβ1-6Galβ1-4GlcNAcβ-sp2 | LeC6'LN |
| 382 | Galβ1-3GalNAcβ1-4Galβ1-4Glcβ-sp3 | aGM1 |
| 383 | Galβ1-4GlcNAcβ1-3Galβ1-4Glcβ-sp4 | LNnT |
| 384 | Galβ1-4GlcNAcβ1-3Galβ1-4GlcNAcβ-sp2 | LN3'LN-C2 |
| 386 | Galβ1-4GlcNAcα1-6Galβ1-4GlcNAcβ-sp2 | LNa6'LN |
| 387 | Galβ1-4GlcNAcβ1-6Galβ1-4GlcNAcβ-sp2 | LN6'LN |
| 388 | Galβ1-4GlcNAcβ1-6(Galβ1-3)GalNAcα-sp3 | LN6TF |
| 389 | GalNAcβ1-3Galα1-4Galβ1-4Glcβ-sp3 | Gb4 |
| 390 | (Glcα1-4)_4_β-sp4 | (Ga4)4b |
| 392 | GalNAcα1-3(Fucα1-2)Galβ1-3GalNAcα-sp3 | A(type 3) |
| 394 | GlcNAcβ1-4(GlcNAcβ1-3)Galβ1-4GlcNAcβ-sp2 | GN2-3',4'LN |
| 395 | GlcNAcβ1-6(GlcNAcβ1-3)Galβ1-4GlcNAcβ-sp2 | Tk |
| 396 | (GlcNAcβ1)_3_-3,4,6-GalNAcα-sp3 | GN3-3,4,6Tn |
| 397 | Galβ1-3GlcN(Fm)β1-3Galβ1-4GlcNAcβ-sp3 | LeC(Fm)b3'LN |
| 398 | Galβ1-3GlcN(Fm)β1-3Galβ1-3GlcNAcβ-sp3 | LeC(Fm)3'LeC |
| 399 | Galβ1-3GlcNAcα1-3Galβ1-3GlcNAcβ-sp2 | LeCa3'LeC-C2 |
| 401 | Galβ1-3GlcNAcβ1-3Galβ1-3GlcNAcβ-sp3 | Le^C^3'Le^C^ |
| 402 | GalNAcα1-3(Fucα1-2)Galα1-4GlcNAcβ-sp3 | Atria4GN |
| 403 | Galβ1-3GlcNAcβ1-3Galβ1-4GlcNAcβ-sp2 | LeC3'LN |
| 404 | GalNAcα1-3Galβ1-4(Fucα1-3)GlcNAcβ-sp3 | ANaLeX |
| 405 | Galα1-3(Fucα1-2)Galα1-4GlcNAcβ-sp3 | Btria4GN |
| 406 | GalNAcα1-3(Fucα1-2)Galα1-3GalNAcβ-sp3 | Atria3AN |
| 407 | Galβ1-4GlcNAcβ1-3Galβ1-4Glcα-sp4 | LNnTa |
| 408 | GlcNAcβ1-4(GlcNAcβ1-3)Galβ1-4GlcNAcβ-sp3 | GN2-3',4'LN-C3 |
| 419 | 3-O-SuGalβ1-4GlcNAcβ1-3Galβ1-4GlcNAcβ-sp3 | (3'SuLN)3'LN |
| 420 | 4-O-SuGalβ1-4GlcNAcβ1-3Galβ1-4GlcNAcβ-sp3 | (4'SuLN)3'LN |
| 421 | Galβ1-3GlcNAcβ1-3Galβ1-4Glcα-sp4 | LNTa |
| 423 | Neu5Acα2-3Galβ1-4(Fucα1-3)GlcNAcβ-sp3 | SiaLeX |
| 425 | Neu5Acα2-3Galβ1-4(Fucβ1-3)GlcNAcβ-sp3 | bF-SiaLeX |
| 426 | Neu5Acα2-3Galβ1-3(Fucα1-4)GlcNAcβ-sp3 | SiaLeA |
| 428 | Neu5Acα2-3Galβ1-4(Fucα1-3)(6-O-Su)GlcNAcβ-sp3 | SiaLeX6Su |
| 429 | Neu5Acα2-3(6-O-Su)Galβ1-4(Fucα1-3)GlcNAcβ-sp3 | SiaLeX6'Su |
| 431 | Neu5Acα2-3Galβ1-4(2-O-Su-Fucα1-3)GlcNAcβ-sp3 | SiaLeX2'''Su |
| 432 | Neu5Acα2-3Galβ1-4(3-O-Su-Fucα1-3)GlcNAcβ-sp3 | SiaLeX3'''Su |
| 433 | Neu5Acα2-6(Neu5Acα2-3Galβ1-3)GalNAcα-sp3 | Sia2-3',6TF |
| 434 | Neu5Acα2-8Neu5Acα2-3Galβl-4Glcβ-sp4 | GD3 |
| 435 | Neu5Acα2-3Galβ1-4(2-O-Su-Fucα1-3)(6-O-Su)GlcNAcβ-sp3 | SiaLeX6,2'''Su2 |
| 436 | 4-O-Su-Neu5Acα2-3(6-O-Su)Galβ1-4(Fucα1-3)GlcNAcβ-sp3 | SiaLeX6,4''Su2 |
| 437 | GalNAcα1-3(Fucα1-2)Galβ1-3GalNAcβ-sp3 | A(type 4) |
| 438 | Fucβ1-2Galβ1-4(Fucα1-3)GlcNAcβ-sp3 | LeYbF |
| 440 | Neu5Acβ2-6(Fucα1-2)Galβ1-4GlcNAcβ-sp3 | bSia6'Htype2 |
| 441 | Neu5Acα2-6(Fucα1-2)Galβ1-4GlcNAcβ-sp3 | Sia6'Htype2 |
| 479 | Fucα1-2Galβ1-3GlcNAcβ1-3Galβ1-4Glcβ-sp4 | Htype1Lac |
| 480 | Fucα1-2Galβ1-3GlcNAcβ1-3Galβ1-4GlcNAcβ-sp2 | Htype1LN |
| 481 | Galα1-3Galβ1-4GlcNAcβ1-3Galβ1-4Glcβ-sp4 | Galili5 |
| 482 | Galα1-3(Fucα1-2)Galβ1-3(Fucα1-4)GlcNAcβ-sp3 | BLeB |
| 483 | Galα1-3(Fucα1-2)Galβ1-4(Fucα1-3)GlcNAcβ-sp3 | BLeY |
| 484 | GalNAcα1-3(Fucα1-2)Galβ1-3(Fucα1-4)GlcNAcβ-sp3 | ALeB |
| 485 | Galβ1-4GalNAcα1-3(Fucα1-2)Galβ1-4GlcNAcβ-sp3 | Ab4ANa3'(Fa2')LN |
| 489 | Galβ1-4GlcNAcβ1-3(GlcNAcβ1-6)Galβ1-4GlcNAcβ-sp2 | LN3'(GN6')LN |
| 491 | GalNAcα1-3(Fucα1-2)Galβ1-4(Fucα1-3)GlcNAcβ-sp3 | ALeY |
| 492 | (Glcα1-6)_5_β-sp4 | (Ga6)5b |
| 493 | (GlcNAcβ1-4)_5_β-sp4 | Ch5 |
| 495 | Manα1-6(Manα1-3)Manα1-6(Manα1-3)Manβ-sp4 | (Ma)5b |
| 496 | Fucα1-2Galβ1-3(Fuca1-4)GlcNAcβ1-3Galβ1-4Glcβ-sp4 | LeBLac |
| 498 | Galβ1-4GlcNAcβ1-3Galβ1-4GlcNAcβ1-3Galβ1-4GlcNAcβ-sp3 | (LNb3')3 |
| 499 | Galβ1-4GlcNAcβ1-6(Galβ1-4GlcNAcβ1-3)Galβ1-4GlcNAcβ-sp2 | LN2-3',6'LN |
| 501 | Galβ1-3GalNAcβ1-3Galα1-4Galβ1-4Glcβ-sp4 | Gb5 |
| 502 | (Glcα1-6)_6_β-sp4 | (Ga6)6b |
| 503 | (GlcNAcβ1-4)_6_-sp4 | Ch6 |
| 504 | (Aβ1-4GNβ1-2Mα1)_2_-3,6-Mβ1-4GNβ1-4GNβ-sp4 | 9-OS |
| 505 | (GNβ1-2Mα1)_2_-3,6-Mβ1-4GNβ1-4GNβ-sp4 | 7-OS |
| 506 | Ara*f*β1-2Ara*f*α1-5(Ara*f*β1-2Ara*f*α1-3)Ara*f*α1-5Ara*f*α-O(CH_2_)_2_NHCOCH_2_(OCH_2_CH_2_)_6_NH_2_ | Araf6 |
| 507 | GalNAcα1-3GalNAcβ1-3Galα1-4Galβ1-4Glcβ-sp2 | Fs-5 |
| 508 | GalNAcβ1-3(Fucα1-2)Galβ1-4(Fucα1-3)GlcNAcβ-sp3 | ALeYb |
| 509 | Galβ1-3GalNAcβ1-4(Neu5Acα2-3)Galβ1-4Glcβ-sp4 | GM1 |
| 510 | Mana1-6(Mana1-3)Mana1-6(Mana1-3)Mana-sp4 | Man5a |
| 527 | Neu5Acα2-3Galβ1-4GlcNAcβ1-3Galβ1-4GlcNAcβ-sp2 | 3'SLN-LN |
| 528 | Neu5Acα2-3Galβ1-4(Fucα1-3)GlcNAcβ1-3Galβ-sp3 | SiaLeX3A |
| 529 | Neu5Acα2-6(Galβ1-3)GlcNAcβ1-3Galβ1-4Glcβ-sp4 | LSTb |
| 530 | (Neu5Acα2-3Galβ1)_2_-3,4-GlcNAcβ-sp3 | (Sia3A)2-3,4GN |
| 531 | Neu5Acα2-8Neu5Acα2-3(GalNAcβ1-4)Galβ1-4Glcβ-sp2 | GD2 |
| 534 | Neu5Acα2-6Galβ1-4GlcNAcβ1-3Galβ1-4GlcNAcβ-sp3 | 6'SLN-LN |
| 535 | Neu5Acα2-8Neu5Acα2-3(GalNAcβ1-4)Galβ1-4Glcβ-sp4 | GD2-Gly |
| 536 | Neu5Aα2-3Galβ1-3GlcNAcβ1-3Galβ1-4Glcβ-sp4 | LSTa |
| 538 | Galβ1-4(Fucα1-3)GlcNAcβ1-6(Galβ1-3GlcNAcβ1-3)Galβ1-4Glcβ-sp4 | MFLNH III |
| 539 | Galβ1-4GlcNAcβ1-6(Fucα1-2Galβ1-3GlcNAcβ1-3)Galβ1-4Glcβ-sp4 | MFLNH I |
| 540 | Galβ1-4(Fucα1-3)GlcNAcβ1-6(Neu5Acα2-6Galβ1-4GlcNAcβ1-3)Galβ1-4Glcβ-sp4 | MSMFLNnH |
| 541 | Galβ1-4(Fucα1-3)GlcNAcβ1-6(Fucα1-2Galβ1-3GlcNAcβ1-3)Galβ1-4Glcβ-sp4 | DFLNH (a) |
| 542 | Galβ1-3GlcNAcβ1-3Galβ1-4(Fucα1-3)GlcNAcβ1-6(Galβ1-3GlcNAcβ1-3)Galβ1-4Glcβ-sp4 | MF(1-3)iLNO |
| 546 | Neu5Aα2-3Galβ1-4GlcNAcβ1-3Galβ1-4Glcα-sp4 | LSTda |
| 547 | Neu5Aα2-3Galβ1-3GlcNAcβ1-3Galβ1-4Glcα-sp4 | LSTaa |
| 548 | Neu5Aα2-6Galβ1-4GlcNAcβ1-3Galβ1-4GlcNAcβ-sp2 | 6'SLN-LN-C2 |
| 625 | (GlcAβ1-3GlcNAcβ1-4)_11-12_-NH_2_-ol | HyalU(11-12)-ol |
| 627 | (Siaα2-6Aβ1-4GNβ1-2Mα1)2-3,6-Mβ1-4GNβ1-4GNβ-sp4 | 11-OS |
| 630 | (GlcAβ1-3GlcNAcβ1-4)_20_-NH(*p*-C_6_H_4_)CH_2_CH_2_NH_2_ | HyalU20-ol |
| 631 | (GlcAβ1-3GlcNAcβ1-4)_38_-NH(*p*-C_6_H_4_)CH_2_CH_2_NH_2_ | HyalU38-ol |
| 632 | (GlcAβ1-3GlcNAcβ1-4)_13_-NH(*p*-C_6_H_4_)CH_2_CH_2_NH_2_ | HyalU13-ol |
| 633 | (Neu5Acα2-8)_n_-NH(p-C_6_H_4_)CH_2_CH_2_NH_2_ | (Neu5Aca2-8)n |
| 800 | GlcNAcα1-4GlcNAcβ-sp3 | GNa4GN |
| 801 | GalNAcα1-3GalNAc(fur)β-sp3 | Fs(f)-2 |
| 802 | Galβ1-3GalNAc(fur)β-sp3 | Tbb(f) |
| 804 | [Galβ1-4GlcNAcβ-OCH_2_CH_2_]_2_NH | LN_dimer |
| 805 | GalNAcβ1-4(6-O-Bn)GlcNAcβ-sp3 | 6'Bn-LacdiNAc |
| 806 | Galα1-6Glcα-sp3 | Aa6Ga |
| 808 | Galα1-6Glcβ-sp3 | Aa6G-C3 |
| 809 | GalNAcβ1-3GalNAcα-sp3 | ANb3ANa |
| 810 | GalNGcα1-3GalNAcα-sp3 | core5Gc |
| 811 | 3,6-O-Me_2_-Glcβ1-4(2,3-O-Me_2_)Rhaβ-O(*p*-C_6_H_4_)-OCH_2_CH_2_NH_2_ | KN05097 |
| 812 | Galβ1-4Glcα-sp4 | Laca |
| 813 | Galβ1-3GalNGcα-sp3 | TFGc |
| 814 | Manα1-6Manα-sp4 | Ma6Ma |
| 815 | Galα1-4GalNAcα-sp3 | Aa4ANa |
| 816 | Galβ1-4GalNAcα-sp3 | Ab4ANa |
| 817 | GalNAcβ1-4GalNAcα-sp3 | ANb4ANa |
| 818 | GalNAcα1-4GalNAcα-sp3 | ANa4ANa |
| 819 | Glcβ1-4GalNAcα-sp3 | G4ANa |
| 820 | GlcNAcβ1-4GalNAcα-sp3 | GN4ANa |
| 821 | Galα1-4Galβ-sp3 | Aa4A |
| 822 | GalNAcα1-4Galβ-sp3 | ANa4A |
| 823 | GalNAcβ1-4Galβ-sp3 | ANb4A |
| 824 | Galβ1-4Galβ-sp3 | Ab4A |
| 850 | Galβ1-3(6-O-Su)GalNAcα-sp3 | 6SuTF |
| 851 | Galα1-3(6-O-Su)GalNAcα-sp3 | 6SuTaa |
| 852 | GlcNAcβ1-4-[HOOC(CH_3_)CH]-3-O-GlcNAcα-sp4 | GN-aMur |
| 853 | 6-O-Su-Galα1-3GalNAcα-sp3 | 6'SuTaa |
| 854 | Neu5Acα2-3(6-O-Su)Galβ-sp3 | Sia3A6'Su |
| 855 | (O-Su)_4_Glcα1-4(O-Su)_3_Glcβ-spacer | MaltSu7 |
| 1001 | -4Qui3Nα1-3Rhaα1-4Galβ1-3(Glcβ1-4)GalNα1- | S. enterica O28deAc |
| 1002 | -2Rib-ol5-P-6Galα1-3FucNAmα1-3GlcNβ1- | S. enterica O47deAc |
| 1003 | -4(Fucα1-3)GalNα1-6ManNα1-3Fucα1-3(Glcβ1-4)Galβ1- | S. enterica O16deAc |
| 1004 | -2Fucα1-2Galβ1-3GalNAcα1-3GlcNAcα1- | S. enterica O13 |
| 1005 | -4Qui3NAcα1-3Rhaα1-4Galβ1-3(Glcβ1-4)GalNAcα1- | S. enterica O28 |
| 1006 | -4(Fucα1-3)GalNAcα1-6Man2(20%)Ac3(40%)Ac4(20%)Acα1-3Fucα1-3(Glcβ1-4)Galβ1- | S. enterica O16 |
| 1007 | -2(Gal*f*α1-4)Galα1-3ManNAcβ1-6Gal*f*β1-3GlcNAcβ1- | S. enterica O17 |
| 1008 | -3Gal*f*2(30%)Acβ1-3Galα1- | S. enterica O67 |
| 1009 | -3(S-3HOBut1-2Ala1-4)Qui4Nβ1-6GlcNAcα1-3QuiNAcα1-3GlcNAcα1- | S. enterica O58 |
| 1010 | -2Manβ1-4Glcα1-3QiuNAcα1-3GlcNAcα1- | S. enterica O41 |
| 1011 | -3(GalNAcA6NH_2_α1-2)Rhaα1-2Rhaα1-3Rhaα1-2Rhaα1-3GlcNAcβ1- | S. enterica O62 |
| 1012 | -2(Fuc3NFoα1-3)Manβ1-3Glcβ1-3GlcNAcβ1- | S. enterica O60 |
| 1013 | -4Manα1-2Manα1-2Manβ1-3GalNAcα1- | S. enterica O18 |
| 1014 | -2Galβ1-3GlcNAcα1-4Rhaα1-3GlcNAcβ1- | S. enterica O59 |
| 1015 | -3(ManNAcβ1-2)Rhaα1-2Rhaα1-2Galα1-3GlcNAcβ1- | S. enterica O42 |
| 1016 | -2Rib*f*β1-4Galβ1-4GlcNAcα1-4Galβ1-3GlcNAcα1- | S. enterica O52 |
| 1017 | -3GlcNAcβ1-3(Manβ1-4)Galα1-4Rhaα1- | S. enterica O11 |
| 1018 | -6Glcα1-4(GlcNAcβ1-3)Galβ1-3GalNAcα1-3GlcNAcβ1- | S. enterica O51 |
| 1019 | -2Glcα1-6Glcα1-4(GlcNAcβ1-3)Galα1-3GlcNAcβ1- | S. enterica O44 |
| 1020 | -7Neu5Acα2-3FucNAmα1-3GlcNAc6Acβ1- | S. enterica O21 |
| 1021 | -3(GlcNAcβ1-2)Rhaα1-2Rhaα1-4Glcα1-3GalNAcβ1- | S. enterica O57 |
| 1022 | -3(Ser2Ac1-4)Qui4Nβ1-3Rib*f*β1-4GalNAcα1-3GlcNAcα1- | S. enterica O56 |
| 1023 | -3(Galβ1-4)Galβ1-4(GlcNAcβ1-2)Glcβ1-3GalNAcβ1- | S. enterica O38 |
| 1024 | -3(Rha2(%)Ac3(%)Ac4(%)Acα1-4GalAα1-2)Rhaα1-4Glcα1-2Rhaα1-3GlcNAcβ1- | C. sakazakii G2356 O2 |
| 1025 | -3FucNAcα1-4(GlcNAcβ1-2)GalAα1-3FucNAcα1-3GlcNAcβ1- | C. sakazakii G2592 O7 |
| 1026 | -2(Glcα1-4)Glcβ1-2Fuc3N*R*Hbβ1-6GlcNAcα1-4GalNAcα1-3(Glcα1-6)GlcNAcβ1- | C. sakazakii G2594 O4 |
| 1027 | -4Qui3NAcα1-3Rhaα1-6GlcNAcα1-4GlcAβ1-3(Glcβ1-4)GalNAcα1- | C. sakazakii G2726 O3 |
| 1201 | -4(Colα1-3)(Colα1-6)Glcα1-4Galα1-3GlcNAcβ1- | E. coli O11 |
| 1202 | -2Galβ1-3FucNAcα1-3GlcNAcβ1- | E. coli O15 |
| 1203 | -2Manα1-2(Glcα1-4)Manβ1-3GlcNAcα1-6Manα1- | E. coli O44 |
| 1204 | -2(S-3HOBut1-4)Qui4Nα1-4GalNAcβ1-4Rhaα1-3GlcNAc6(30%)Acβ1- | E. coli O49 |
| 1205 | -3(GlcNAcβ1-2)Rhaα1-2Rhaα1-4Glcα1-3GalNAcβ1- | E. coli O51 |
| 1206 | -3Fuc*f*2(50%)Acβ1-3-6dmanHepβ1- | E. coli O52 |
| 1207 | -2-DRha4NAcα1-3Fucα1-4Glcβ1-3GalNAcα1- | E. coli O57 |
| 1208 | -4(R-Lac2-3Rhap2Acα1-3)Manβ1-4Manα1-3GalNAcβ1- | E. coli O58 |
| 1209 | -4Quip3NAcα1-3Rhaα1-4Galβ1-3GalNAcα1- | E. coli O71 |
| 1210 | -6Manα1-2(Glcα1-4)Manα1-2(Glcα1-3)Manβ1-3GlcNAcα1- | E. coli O73 |
| 1211 | -2(Gal*f*α1-4)Galα1-3ManNAcβ1-6Gal*f*β1-3GlcNAcβ1- | E. coli O85 |
| 1212 | -3)Fucα1-3Xlu*f*β1- | E. coli O95 |
| 1213 | -3(Glcα1-2)Rhaα1-2Rhaα1-2Rhaα1-3(Glcα1-2)Rhaα1- | E. coli O99 |
| 1214 | -4-8eLeg5Ac7Acα2-6Galα1-3FucNAcα1-3GlcNAcα1- | E. coli O108 |
| 1215 | -4(GlcpNAcβ1-3)GalNAcα1-2Glcα1-4L-IdoAα1-3GalNAcβ1- | E. coli O112ab |
| 1216 | -3Rib-ol5-P-6Galα1-3FucNAmα1-3GlcNAcβ1- | E. coli O118 |
| 1217 | -2(RhaNAc3NFoβ1-3)Manβ1-3Galα1-4Rhaα1-3GlcNAcα1- | E. coli O119 |
| 1218 | -3(S-3HOBut1-2DAla1-4)Qui4Nβ1-6GlcNAcα1-3LQuiNAcα1-3GlcNAc6(30%)Acα1- | E. coli O123 |
| 1219 | -2(Glcα1-3)Manα1-3Fucα1-3GalNAcα1-4(Galβ1-3)GalNAcβ1- | E. coli O125 |
| 1220 | -2Fuc3(65%)Ac4(35%)Acα1-2Galβ1-3GalNAcα1-3GalNAcα1- | E. coli O127 |
| 1221 | -4(GalNAcβ1-3)Galα1-6Glcβ1-3GalNAcβ1- | E. coli O130 |
| 1222 | -3Rhaα1-3Rhaα1-2Glcα1-3GlcNAcα1- | E. coli O148 |
| 1223 | -3(S-Lac2-4)GlcNAcβ1-2Rhaα1-2Rhaα1-3(Glcβ1-2)Rhaα1-3GlcNAcβ1- | E. coli O150 |
| 1224 | -2Rib-ol5-P-6Galα1-3FucNAmα1-3(GlcNAcβ1-4)GlcNAcβ1- | E. coli O151 |
| 1225 | -8(D-Ala1-7)Leg5Acα2-4GlcAβ1-3GlcNAcβ1- | E. coli O161 |
| 1226 | -4(Fucα1-3)GlcNAc6(30%)Acα1-4GlcAα1-3Fucα1-3GlcNAcβ1- | E. coli O168 |
| 1227 | -2Galβ1-4Manβ1-4Galα1-3GlcNAcβ1- | E. coli O40 |
| 1228 | -2)Galα1-3(Fucα1-2)Galβ1-3GalNAcβ1-3GalNAcβ(1- | E. coli O86_B7 |
| 1230 | Escherichia coli O10a10b | E. coli O10a10b |
| 1231 | -2Glcβ1-6GlcNAcα1-3FucNAcα1-3GlcNAcβ1- | E. coli O12 |
| 1232 | Galα1-2Galα1-2(Galβ1-4)Glcα1-3Glcα1-/inner core-lipid A/ | E. coli O14 |
| 1233 | -2Rhaα1-2Rhaα1-2Rhaα1-2Glcα1-3GlcNAc6Acα1- | E. coli O19ab |
| 1234 | Escherichia coli O27 | E. coli O27 |
| 1235 | -4(Rhaα1-2Fucα1-3)Manα1-3Fucα1-3GlcNAcβ1- | E. coli O36 |
| 1236 | -4(D-Gro1-*P*-O-3)GalNAcβ1-3Galα1-4Galβ1-3GalNAcβ1- | E. coli O37 |
| 1237 | -3(R-3HOBut1-4)Qui4Nβ1-4(Galα1-3)Manα1-4Rhaα1-3GlcNAcα- | E. coli O39 |
| 1238 | -3Galα1-3(GlcAβ1-4)Fucα1-4GlcNAcβ1-3Fucα1-3GlcNAcβ1- | E. coli O41 |
| 1239 | Escherichia coli O54 | E. coli O54 |
| 1240 | Escherichia coli O62 | E. coli O62 |
| 1241 | Escherichia coli O81 | E. coli O81 |
| 1242 | -4GlcAβ1-4(GlcNAcβ1-2)GlcAβ1-3GlcNAcα1- | E. coli O30 |
| 1243 | -4(Rhaα1-2Fucα1-3)Manα1-3Fucα1-3GlcNAcβ1- | E. coli O36 |
| 1244 | Escherichia coli O37 | E. coli O37 |
| 1245 | -2Galβ1-4Manβ1-4Galα1-3GlcNAcβ1- | E. coli O40 |
| 1246 | -4(D-aThr3(70%)Ac2-6)GlcAβ1-6Galβ1-6Glcβ1-3GalNAc6(15%)Acβ1- | E. coli O46 |
| 1247 | -6(Rhaα1-3)Manα1-2(Glcα1-3)Manα1-2Manα1-2Manβ1-3GlcNAcα1- | E. coli O68 |
| 1248 | GlcNAcα1-2Glcα1-2Glcα1-3(Galα1-6)Glcα1-/inner core-lipid A/ | E. coli O100 |
| 1249 | Escherichia coli O102 | E. coli O102 |
| 1250 | -3(Rhaα1-4)GlcAα1-2Rhaα1-2Rhaα1-2Galα1-3GalNAcβ1- | E. coli O120 |
| 1251 | -2Rhaα1-2Rhaα1-3Rha2Acα1-3(Glcα1-6)GlcNAcβ1- | E. coli O135 |
| 1252 | -3(Gal*f*α1-2Rhaα1-4)Galβ1-4Glcα1-4GlcAα1-3GalNAcβ1- | E. coli O140 |
| 1253 | -2Rib*f*β1-4Galβ1-4GlcNAcα1-4Galβ1-3GlcNAcα1- | E. coli O153 |
| 1254 | -2(ManNAcα1-3)Rhaα1-3Rhaα1-3Rhaα1-3GalNAcα1- | E. coli O154 |
| 1255 | -2Rha4NAcα1-3Fucα1-4Glcβ1-3GalNAcα1- | E. coli O157 |
| 1256 | -4(Rhaα1-3)(Glcα1-6)Glcα1-3GalNAcα1-3GalNAcβ1- | E. coli O158 |
| 1257 | -2Manβ1-4GlcAβ1-3LQuiNAcα1-3GlcNAcα1- | E. coli O163 |
| 1258 | -4ManNAc3NAcAβ1-2Rhaα1-3Rhaβ1-4GlcNAcα1- | E. coli O180 |
| 1259 | Escherichia coli O84-deAc | E. coli O84deAc |
| 1301 | -3(Qui3NFoα1-4)GalA6NH2α1-4GalNAcα1-4Galα1-3GalNAcβ1- | P. alcalifaciens O3 |
| 1302 | Providencia alcalifaciens O3_2 capsular polysaccharide | P. alcalifaciens O3 CPS |
| 1303 | -4GlcNAcβ1-3GlcAα1-4GlcNAcα1-3Rha2Acβ1- | P. alcalifaciens O7 |
| 1304 | -2Glcβ1-6Galα1-6GalNAcα1-4(Glcβ1-3)GalNAcα1-3GalNAcα1- | P. alcalifaciens O9 |
| 1305 | Providencia rustigianii O11 capsular polysaccharide | P. rustigianii O11 CPS |
| 1306 | -4(GlcNAcβ1-2Glcβ1-2)(GlcNAcβ1-3)Galβ1-3GalNAcα1-4Galβ1-3GalNAcβ1- | P. alcalifaciens O12 |
| 1307 | -4(D-GroA1NH2(2-P-3))GalNAcβ1-4Galβ1-3FucNAc4Nβ1- | P. alcalifaciens O22 |
| 1308 | -3(Dhpa2-4Manβ1-4)Galα1-4GalNAcβ1-3GalNAcβ1- | P. alcalifaciens O31 |
| 1309 | -4Qui3NFoβ1-3Galα1-3GlcAβ1-3GalNAcβ1- | P. alcalifaciens O40 |
| 1310 | -3GlcAβ1-4(Glcα1-3)Fucα1-4Fucα1-2Glcβ1-3GlcNAcα1- | P. alcalifaciens O46deAc |
| 1311 | -3Manα1-2Fucα1-2GlcA4Acβ1-3GalNAcα1- | P. alcalifaciens O48 |
| 1312 | -4Glcβ1-3Galα1-4GalNAcβ1-4(L-Ser2-6)GlcAβ1-3GalNAcβ1- | P. alcalifaciens O60 |
| 1401 | GalNAcα1-4GlcNAc3NAcAβ1-3DFucNAcα1-3QuiNAcβ1-3Rhaα1-3(Glcα1-6)Glcβ1-3(Glcα1-4)(L-Ala1-2)GalNα(P-6)1-3L-gro-manHepp7Cmα1-3(P-2)(P-4)L-gro-manHepα1-5Kdoα | P. aeruginosa O1(F4) |
| 1402 | -3Rhaα1-4LGalNAcAα1-3QuiNAcα1- | P. aeruginosa O10ac(F5) |
| 1403 | -2LGlcβ1-3FucNAcα1-3DFucNAcβ1- | P. aeruginosa O11ab |
| 1404 | -2Rhaα1-3Rhaα1-4GalNAcA3Acα1-3QuiNAcβ1- | P. aeruginosa O13ab |
| 1405 | Rhaα1-3Rhaα1-4GalNAcAα1-3QuiNAcβ1-3Rhaα1-3(Glcα1-6)Glcβ1-3(Glcα1-4)(L-Ala1-2GalNα1-3(P-6)L-gro-manHep7Cmα1-3(P-2)(P-4)L-gro-manHepα1-5Kdoα | P. aeruginosa O13ac |
| 1406 | Glcα1-6Glcβ1-3(Rhaα1-6Glcα1-4)GalNα1-3(P-6)L-gro-manHepα1-3(P-2)(P-4)L-gro-manHepα1-5(Kdoα2-4)Kdoα2-6(P-4)GlcNβ1-6GlcNα1-P | P. aeruginosa O14 |
| 1407 | -2Rib*f*β1-3GalNAcα1- | P. aeruginosa O15 |
| 1408 | Pseudomonas aeruginosa O2abc | P. aeruginosa O2abc |
| 1409 | -4ManNAc3NAmAβ1-4LGulNAc3NAcAα1-3DFucNAc4Nβ1- | P. aeruginosa O2ac |
| 1410 | -4ManNAc3NAmAβ1-4LGulNAc3NAcAα1-3DFucNAcβ1- | P. aeruginosa O2ac(F3) |
| 1411 | -4ManNAc3NAmAβ1-4ManNAc3NAcAβ1-3DFucNAcα1- | P. aeruginosa O2ad(F7) |
| 1412 | -4LGulNAc3NAmAα1-4ManNAc3NAcAβ1-3DFucNAc4Acα1- | P. aeruginosa O2adf |
| 1413 | -2LRha3Acα1-6GlcNAcα1-4LGalNAcA3Acα1-3(S-3HOBut1-4)QuiNAc4Nβ1- | P. aeruginosa O3(Habs 3) |
| 1414 | -2Rha3Acα1-6GlcNAcα1-4LGalNAcAα1-3QuiNAc4NSHbβ1- | P. aeruginosa O3ab |
| 1415 | -2Rhaα1-3FucNAcα1-3FucNAcα1-3QuiNAcα1- | P. aeruginosa O4ab |
| 1416 | -2Rhaα1-3FucNAcα1-3FucNAcα1-3DFucNAcα1- | P. aeruginosa O4ac |
| 1417 | -2Rhaα1-4GalNAcA3Acα1-4GalNFoAα1-3QuiNAcα1- | P. aeruginosa O6(F1) |
| 1418 | -3R-3HOBut1-7Pse4Ac5Acβ2-4DFucNAcα1-3QuiNAcβ1- | P. aeruginosa O9ad |
| 1501 | Proteus genomospecies 5/6 O79 | P. genomospecies 5/6 O79 |
| 1502 | -3GalNAcβ1-4(L6dTalα1-3)Manα1-3L6dTalα1- | A. hydrophila O34deAc |
| 1503 | -2Rhaα1-2Rhaα1-2Rhaα1-4GalAα1-3GlcNAcα1- | E. cloacae G2277 |
| 1504 | -3Rhaβ1-4(Glcα1-3)Rhaα1-2Rhaα1-3Galα1-3DFucNAcα1- | E. cloacae G3421 |
| 1601 | Proteus mirabilis 12B-r | P. mirabilis 12B-r |
| 1602 | -3(Rib1(50%)Ac-ol5-P-6)Galβ1-4(GlcNAcβ1-2)Glcβ1-3GlcNAcβ1- | P. mirabilis 1B-m |
| 1603 | -2Glcβ1-3L6dTal2(85%)Acα1-3GlcNAcβ1- | P. mirabilis 3B-m |
| 1604 | -4(LAltpAα1-3)GalNAcα1-3GalAα1-3GlcNAcα1- | P. mirabilis HJ 4320 |
| 1605 | -3(Glcα1-6)GlcNAcβ1-4(GlcNAcβ1-2)GlcAβ1-3(L-Thr2-6)GalAβ1- | P. mirabilis O11 |
| 1606 | -3GlcNAcβ1-3(S,R-CetLys2-6GalAα1-4)Galα1- | P. mirabilis O13 |
| 1607 | GalNAcβ1-4GalNAcα1-3GlcNAcα1-2Rib-ol | P. mirabilis O16 |
| 1608 | -3GlcAβ1-4(Galα1-3)FucNAca1-3GlcNAcα1- | P. mirabilis O23 |
| 1609 | -4(Lys2-6)GalAα1-4Galα1-3(Ser-(2-6)GalA4Acα1-3GlcNAcβ1- | P. mirabilis O28 |
| 1610 | -3LQuiNAcα1-3GlcNAcα1-6(S-Lac-1-3)GlcNAcα1- | P. mirabilis O31 |
| 1611 | -2(Rib-ol5-P-3)Galβ1-3GlcNAcα1-3(EtN1-75%P-6)Glcβ1-3GlcNAcβ1- | P. mirabilis O33 |
| 1612 | -2Fuc3N(R-3HOBu)4Acβ1-6Glc3Acα1-4GlcAβ1-3GlcNAcα1- | P. mirabilis O35 |
| 1613 | -3(EtNAc1-P-6)GlcNAcα1-3D-Asp2Ac4-4)Qui4Nβ1-6Glcα1-4GalAα1- | P. mirabilis O38 |
| 1614 | -6(GalA6(L-Lys)α1-4)GalNAcβ1-4(Glcα1-2)GlcAβ1-3GalNAcβ1- | P. mirabilis O3ab |
| 1615 | -4(GalA6(L-Thr)3Acα1-3)GalNAcβ1-3Rhaβ1-4GlcNAc6Acβ1- | P. mirabilis O58 |
| 1616 | -4(GlcAα1-3)FucNAcα1-3GlcNAcβ1- | P. mirabilis O6 |
| 1617 | -4(S,R-CetLys2-6)GlcAβ1-6GalNAcα1-6GlcNAcβ1-3GlcNAcβ1- | P. mirabilis O60 |
| 1618 | -6GlcNAcα1-3Galβ1-3GalNAcα1- | P. mirabilis OE |
| 1701 | -3GlcAβ1-4(Galα1-3)FucNAcα1-3GlcNAcα1- | P. penneri 107 |
| 1702 | -3(Glcβ1-3GlcNAc4(S-Lac)β1-2)Rhaα1-2Rhaα1-2Gal6Acα1-3GlcNAcβ1- | P. penneri 113 |
| 1703 | -4(Glca1-2)GlcA3Acβ1-3GlcNAcα1-2(R-3HOBut1-3)Fuc3Nβ1-6Glc4Acα1- | P. penneri 17 |
| 1704 | -6GlcNAc3(S-Lac)α1-3LQuiNAcα1-3GlcNAcα1- | P. penneri 28 |
| 1705 | -3Galα1-4GalNAcα1-3FucNAcα1-3(EtN1-P-6)GlcAcβ1- | P. penneri 31 |
| 1706 | -6(S-Lac2-3)GlcNAcβ1-3Galα1-3GlcNAc6Acβ1- | P. penneri 40 |
| 1707 | -4(Glcα1-3)Glcβ1-3Galβ1-3GalNAcβ1-4Rib-ol5-P- | P. penneri 75 |
| 1801 | -3(EtN1-P-6)GlcNAcα1-2(R-3HOBut1-3)Fuc3Nβ1-6Glcα1-4GlcAβ1- | P. vulgaris 32/57 O17 |
| 1802 | -4(L-Ala2-6)GlcAβ1-3GalNAcβ1-4Glcβ1-3Galα1-4GalNAcβ1- | P. vulgaris 70/57 O44 |
| 1803 | -4GalN6Acα1-3DFuc2Acα1-3(EtN1-P-6)GlcNAcβ1-3Galα1- | P. vulgaris O19ab |
| 1804 | -3GlcNAcβ1-3(Qui3NAc2(65%)Ac4Acα1-2)Rhaβ1-4Rhaα1-4GlcAβ1- | P. vulgaris O22 |
| 1806 | -4GlcAβ1-3GlcNAcβ1-2(R-3HOBut1-2L-Ala1-4)Qui4Nβ1-3Galα1- | P. vulgaris O4 |
| 1807 | -4Glc6(65%)Acα1-3GlcA4Acβ1-3GlcNAcα1-3GlcA4(87%)Acβ1- | P. vulgaris O46 |
| 1808 | -4Glcβ1-3GalNAcβ1-4GalNAcβ1-4Galβ1- | P. vulgaris O65 |
| 1809 | -4LQuiNAcα1-3GlcNAcα1-4(LQuiNAcα1-3)GalNAcα1-4Galα1-P | P. vulgaris OX19 |
| 1810 | -4Glcβ1-3GalNAcβ1-4GalNAcβ1-4Galβ1- | P. vulgaris TG251 |
| 2001 | -3(Rib*f*β1-4GlcAβ1-4)Galα1-6Manα1-2Manα1-3GalNAcβ1- | Sh. boydii type 10 |
| 2002 | -3GlcNAcβ1-4GlcA3Acβ1-2(Rha3Acα1-3)Manα1-4Galβ1- | Sh. boydii type 12 |
| 2003 | -6Galα1-4GlcAβ1-6Galβ1-4Galβ1-4GlcNAcβ1- | Sh. boydii type 14 |
| 2004 | -4(GlcNAcβ1-3)GalNAcα1-4Glcα1-4L-IdoAα1-3GalNAcβ1- | Sh. boydii type 15 |
| 2005 | -4GlcAβ1-2(Galα1-3)Man6(50%)Acβ1-4Manβ1-3GlcNAcβ1- | Sh. boydii type 16 |
| 2006 | -6(R-Lac2-4)Glcβ1-4GalNAcα1-3GalNAcβ1- | Sh. boydii type 17 |
| 2007 | -3Rhaβ1-4Rhaα1-2Rhaα1-2GalAα1-3GalNAcα1- | Sh. boydii type 18 |
| 2008 | -2Rib*f*β1-4GalAα1-3GlcNAcα1-2(Gal*f*β1-3)Rhaα1-2Rhaα1-2Rib*f*β1-4GalAα1- | Sh. boydii type 2 |
| 2009 | -3(GlcAβ1-4)Galα1-6Manα1-2Manα1-3GalNAcβ1- | Sh. boydii type 6 |
| 2010 | -2Gal*f*β1-3GlcNAcα1-8(3HOBut1-7)Pse5Ac2-6Galα1-6Glcα1- | Sh. boydii type 7 |
| 2011 | -3GalNAcα1-4GlcAβ1-3GlcNAcβ1-2GalAβ1- | Sh. boydii type 8 |
| 2012 | -4Glcα1-4GlcAβ1-3GlcNAcα1-3Rhaα1- | Sh. boydii type 9 |
| 2013 | Shigella boydii type X | Sh. boydii type X |
| 2101 | -3Rhaα1-3Rhaα1-2Galα1-3GlcNAcα1- | Sh. dysenteriae type 1 |
| 2102 | -1D-Gro3-P-6Glcβ1-4(Glcα1-6Gal2(25%)Acα1-3)FucNAcα1-3GlcNAcβ1- | Sh. dysenteriae type 11 |
| 2103 | -3(R-Lac2-4Glcβ1-6Glcα1-4)Galβ1-6Gal*f*β1-3GalNAcβ1- | Sh. dysenteriae type 3 |
| 2104 | -3GlcNAcα1-3(Fuc3Ac4Acα1-4)GlcNAcα1-4GlcAα1-3Fucα1- | Sh. dysenteriae type 4 |
| 2105 | GalNAcA3Ac6NH2α1-4GalNAcAα1-3GlcNAc | Sh. dysenteriae type 7 |
| 2106 | -4GlcAβ1-3GalNAcβ1-3(GlcNAcβ1-4Glcβ1-4)GalNAcβ1- | Sh. dysenteriae type 8 |
| 2107 | -2Gal3,4(*R*Pyr)β1-4Manβ1-4Galα1-3GlcNAcβ1- | Sh. dysenteriae type 9 |
| 2201 | -3GlcNAcβ1-2Rhaα1-2Rhaα1-3(Glcα1-4)Rhaα1- | Sh. flexneri type 2a |
| 2202 | -3GlcNAcβ1-2(Glcα1-3)Rhaα1-2Rhaα1-3(Glcα1-4)Rhaα1- | Sh. flexneri type 2b |
| 2203 | -3GlcNAcβ1-2(Glcα1-3)Rhaα1-2Rhaα1-3Rha2Acα1- | Sh. flexneri type 3a |
| 2204 | -2Rhaα1-2Rhaα1-3Rha2Acα1-3GlcNAcβ1- | Sh. flexneri type 3b |
| 2205 | -3(Glcα1-6)GlcNAcβ1-2Rhaα1-2Rhaα1-3Rhaα1- | Sh. flexneri type 4a |
| 2206 | -3(Glcα1-6)GlcNAcβ1-2Rhaα1-2Rhaα1-3Rha2Acα1- | Sh. flexneri type 4b |
| 2207 | -3GlcNAcβ1-2Rhaα1-2(Glcα1-3)Rhaα1-3Rhaα1- | Sh. flexneri type 5a |
| 2208 | -3GlcNAcβ1-2(Glcα1-3)Rhaα1-2(Glcα1-3)Rhaα1-3Rhaα1- | Sh. flexneri type 5b |
| 2209 | -2Rha3(%)Ac4(%)Acα1-2Rhaα1-4GalAβ1-3GalNAcβ1- | Sh. flexneri type 6 |
| 2210 | -2Rha3(60%)Ac4(30%)Acα1-2Rhaα1-4GalAβ1-3GalNAcβ1- | Sh. flexneri type 6b |
| 2211 | -3GlcNAcβ1-2(Glcα1-3)Rhaα1-2Rhaα1-3Rhaα1- | Sh. flexneri type X |
| 2212 | -2Rhaα1-2Rhaα1-3Rhaα1-3GlcNAcβ1- | Sh. flexneri type Y |
| 2213 | -3Rhaa1-3(Glcα1-4)GlcNAcβ1-2Rhaα1-2Rhaα1- | Sh. flexneri type 1a |
| 2214 | Shigella flexneri type 2c | Sh. flexneri type 2c |
| 2215 | -2(EtN1-P-3)Rhaα1-2Rhaα1-3Rhaα1-3(Glcα1-6)GlcNAcβ1- | Sh. flexneri 4av |
| 2216 | Shigella flexneri type 5c | Sh. flexneri type 5c |
| 2217 | -2(Glcα1-3)Rhaα1-2Rhaα1-3Rhaα1-3(Glcα1-4)GlcNAcβ1- | Sh. flexneri type 1d |
| 2218 | Shigella flexneri type Ya | Sh. flexneri type Ya |
| 2219 | -2Rha3(%)Acα1-2Rhaα1-3Rhaα1-3GlcNAc6(%)Acβ1- | Sh. flexneri type Y_2 |
| 2220 | -2(EtN1-P-3)Rhaα1-2(20%EtN1-P-3)Rhaα1-3Rhaα1-3GlcNAc6(45%)Acβ1- | Sh. flexneri type Yv |
| 2221 | -2(Glcα1-3)Rhaα1-2(EtN1-P-3)Rhaα1-3Rhaα1-3GlcNAcβ1- | Sh. flexneri type Xv |
| 2501 | Streptococcus equi sp. hyaluronic acid sodium salt | S. equi sp. hyaluronic acid |
| 2502 | -4(2-O-Su)IdoAα1-4(6-O-Su)GlcNSuα1-; -4GlcAβ1-4GlcNAcα1- | Heparin |
| 3001 | -3Glcβ1- | S. cerevisiae zymozan A |
| 3002 | -6Manα1- | Mannan |
| 3301 | -3Glcβ1-6Glcβ1- | Laminarin |
| 3302 | -3Glcβ1- | Laminaran |
| 3401 | -4Glcβ1-4Glcβ1-3Glcβ1- | bGlucan |
| 3501 | Rha:Ara:Gal:GalA 9:3:79:9, MW 900-2000 kDa | Galactan-1 |
| 3502 | Rha:Ara:Gal:GalA 17:3:62:18, MW 100-400kDa | Galactan-2 |
| 8001 | -2)Galα1-3(Fucα1-2)Galβ1-3GalNAcβ1-3GalNAcβ(1-LPS | E. coli O86_B7 LPS |
| 8002 | -3Rhaα1-3Rhaα1-2Glcα1-3GlcNAcα1- LPS | E. coli O148 LPS |
| 8003 | -4(Fucα1-3)GlcNAc6(30%)Acα1-4GlcAα1-3Fucα1-3GlcNAcβ1- LPS | E. coli O168 LPS |
| 8004 | -4(GalNAcβ1-3)Galα1-6Glcβ1-3GalNAcβ1- LPS | E. coli O130 LPS |
| 8005 | -2Rib-ol5-P-6Galα1-3FucNAmα1-3(GlcNAcβ1-4)GlcNAcβ1- LPS | E. coli O151 LPS |
| 8006 | -3(Glcα1-2)Rhaα1-2Rhaα1-2Rhaα1-3(Glcα1-2)Rhaα1- LPS | E. coli O99 LPS |
| 8007 | -6Manα1-2(Glcα1-4)Manα1-2(Glcα1-3)Manβ1-3GlcNAcα1- LPS | E. coli O73 LPS |
| 8008 | -3Rib-ol5-P-6Galα1-3FucNAmα1-3GlcNAcβ1- LPS | E. coli O118 LPS |
| 8009 | -4(R-Lac2-3Rhap2Acα1-3)Manβ1-4Manα1-3GalNAcβ1- LPS | E. coli O58 LPS |
| 8010 | -4(GlcpNAcβ1-3)GalNAcα1-2Glcα1-4L-IdoAα1-3GalNAcβ1- LPS | E. coli O112ab LPS |
| 8011 | -3(S-Lac2-4)GlcNAcβ1-2Rhaα1-2Rhaα1-3(Glcβ1-2)Rhaα1-3GlcNAcβ1- LPS | E. coli O150 LPS |
| 9001 | -3Glcβ1-3Glcβ1-3(Glcβ1-6)Glcβ1- | Scleroglucan |
| 9002 | -3Glcβ1- | Curdlan |
| 9003 | -4GlcN(%)Acβ1- | Chitosan |
|  |  |  |
| LEGEND: |  |  |
| sp1 = ol = aminoalditol | |  |
| sp2 = C2 = -O(CH_2_)_2_NH_2_ | |  |
| sp3 = C3 = -O(CH_2_)_3_NH_2_ | |  |
| sp4 =Gly = -NHCOCH_2_NH_2_ | |  |
| sp5 =C8 = -O(CH_2_)_3_NH-CO(CH_2_)_5_NH_2_ | |  |
| sp6 = NH(*p*-C_6_H_4_)CH_2_CH_2_NH_2_ (as-aminoalditol) | |  |
| sp7 = Ph = -OC_6_H_4_-p-NH_2_ | |  |
| sp8 = PEG = -(OCH_2_CH_2_)_6_NH_2_ | |  |
| sp9 = Bn = -OCH_2_C_6_H_4_-p-NHCOCH2NH2 | |  |
| sp10 = PEG2 = -(OCH_2_CH_2_)_2_NH_2_ | |  |
| sp11 = -OCH_2_CH_2_CH_2_SCH_2_CH_2_NH_2_ | |  |
| sp12 = PEG3 = H_2_N-(CH_2_CH_2_O)_3_-CH_2_CH_2_CO | |  |
| LPS - lipopolysaccharide | |  |
| sp0 = other spacers:Asn, Ser, C2Et (see details in the column "Spacered form of saccharide") | | |
| Ala = alanine | |  |
| Arg = arginine | |  |
| Asn = asparagine, NH-CO-CH_2_CH(COOH)NH_2_ | |  |
| Ile = iso-leucine | |  |
| Nle = norleucine | |  |
| Ser = serine, -OCH_2_CH(COOH)NH_2_ | |  |
| Trp = tryptophan | |  |
| Phe = phenylalanine, -COCH(CH_2_C_6_H_5_)NH_2_ | |  |
| C2Et = -O(CH_2_)_2_NHEt | |  |
| Cit = citrullin | |  |
| A = Gal |  |  |
| AN = GalNAc | |  |
| Ch = chito |  |  |
| F = L-Fuc |  |  |
| G = Glc |  |  |
| Gc = glycolyl | |  |
| GN = GlcNAcb | |  |
| R = Rha |  |  |
| i = iso |  |  |
| (fur, f) = furanose cycle | |  |
| Fm = formyl | |  |
| Lac = lactose | |  |
| LN = N-acetyllactosamine | |  |
| M = Man |  |  |
| MN = ManNAc | |  |
| Malt = maltose | |  |
| OS = oligosaccharide | |  |
| P = phosphate | |  |
| S = Sia = Neu5Aca | |  |
| Su = sulfate | |  |
| Tn = GalNAca | |  |
| U = uronic acid | |  |
| D-Fuc3N*R*Hb = 3,6-dideoxy-3-[(*R*)-3-hydroxybutanoyl]-D-galactose | |  |
| D-Xlu = D-*threo*-pent-2-ulose (D-xylulose) | |  |
| 8eLeg = 8-epilegionaminic acid | |  |
| 8-Leg = 8-legionaminic acid | |  |
| L-Ido = L-iduronic acid | |  |
| D-aThr = D-allothreonine | |  |
| Dhpa = (2R,4R)-2,4-dihydroxypentanoic acid | |  |
| L-6dTal = 6-deoxy-L-talose | |  |
