# Supplementary material for: Epithelial apical glycosylation changes associated with thin endometrium in women with infertility - a pilot observational study
Source: Reprod Biol Endocrinol. 2021 May 15;19:73. doi: 10.1186/s12958-021-00750-z (PMC8122553; doi:10.1186/s12958-021-00750-z)
Supplement: Supplementary file 2 — Additional file 2: Table S2. Glycoconjugates contents on the apical surface of the luminal and glandular epithelial cells in patients with “thin” endometrium (group 1) and patients with normal endometrium (group 2). [file 12958_2021_750_MOESM2_ESM.docx]

Table S2

Glycoconjugates contents on the apical surface of the luminal and glandular epithelial cells in patients with “thin” endometrium (group 1) and patients with normal endometrium (group 2)

|  | group 1 (n=14) | group 2 (n=18) | p- value |
| --- | --- | --- | --- |
| **apical surface of the luminal epithelial cells** | | | |
| **Lectins** | | | |
| MAL II | 0,29±0,07 (0,18-0,41) | 0,28±0,11 (0,14-0,52) | 0,9110 |
| UEA I | 0,24±0,11 (0,06-0,50) | 0,24±0,14 (0,07-0,59) | 0,9325 |
| SNA | 0,32±0,09 (0,14-0,44) | 0,34±0,08 (0,21-0,52) | 0,4953 |
| ECL | 0,35±0,12 (0,11-0,51) | 0,33±0,09 (0,15-0,52) | 0,5973 |
| ECL + N | 0,51±0,11 (0,24-0,68) | 0,57±0,09 (0,39-0,78) | 0,1357 |
| VVL | 0,25±0,10 (0,15-0,46) | 0,26±0,09 (0,10-0,43) | 0,8300 |
| Con A | 0,39±0,09 (0,26-0,53) | 0,35±0,09 (0,15-0,54) | 0,2182 |
| **Antibodies** | | | |
| MECA-79 | **0,33±0,07 (0,25-0,48)** | **0,42±0,11 (0,19-0,65)** | **0,0332** |
| Anti- Le^Y^ | 0,25±0,06 (0,15-0,39) | 0,22±0,06 (0,12-0,35) | 0,1789 |
| **apical surface of the glandular epithelial cells** | | | |
| **Lectins** | | | |
| MAL II | 0,27±0,08 (0,15-0,45) | 0,27±0,08 (0,15-0,45) | 0,9789 |
| UEA I | 0,17±0,10 (0,08-0,41) | 0,22±0,14 (0,05-0,49) | 0,3412 |
| SNA | 0,17±0,07 (0,07-0,29) | 0,19±0,11 (0,06-0,46) | 0,7020 |
| ECL | 0,26±0,10 (0,10-0,42) | 0,29±0,14 (0,08-0,62) | 0,5533 |
| ECL + N | 0,57±0,28 (0,27-1,49) | 0,55±0,10 (0,39-0,72) | 0,8289 |
| VVL | 0,24±0,13 (0,07-0,53) | 0,21±0,09 (0,09-0,38) | 0,4688 |
| Con A | 0,28±0,06 (0,17-0,44) | 0,30±0,07 (0,17-0,40) | 0,5462 |
| **Antibodies** | | | |
| MECA-79 | 0,19±0,07 (0,03-0,29) | 0,20±0,06 (0,10-0,31) | 0,6373 |
| Anti- Le^Y^ | 0,14±0,06 (0,08-0,34) | 0,14±0,06 (0,05-0,27) | 0,9659 |

* Data are presented as mean ± standard deviation (minimum-maximum), t-test

**Bold** statistically significant values
